# Augmented Enterocyte Damage During *Candida albicans* and *Proteus mirabilis* Coinfection

**DOI:** 10.3389/fcimb.2022.866416

**Published:** 2022-05-16

**Authors:** Maria Joanna Niemiec, Mario Kapitan, Maximilian Himmel, Kristina Döll, Thomas Krüger, Tobias G. Köllner, Isabel Auge, Franziska Kage, Christopher J. Alteri, Harry L.T. Mobley, Tor Monsen, Susanne Linde, Sandor Nietzsche, Olaf Kniemeyer, Axel A. Brakhage, Ilse D. Jacobsen

**Affiliations:** ^1^ Leibniz Institute for Natural Product Research and Infection Biology – Hans Knöll Institute, Jena, Germany; ^2^ Center for Sepsis Control and Care, Jena, Germany; ^3^ Department of Biochemistry, Max Planck Institute for Chemical Ecology, Jena, Germany; ^4^ Department of Natural Sciences, University of Michigan-Dearborn, Dearborn, MI, United States; ^5^ Department of Microbiology and Immunology, University of Michigan Medical School, Ann Arbor, MI, United States; ^6^ Department Clinical Microbiology, Umeå University, Umeå, Sweden; ^7^ Center for Electron Microscopy, University Hospital, Jena, Germany; ^8^ Institute of Microbiology, Friedrich Schiller University, Jena, Germany

**Keywords:** *Candida albicans*, *Proteus mirabilis*, coinfection, synergism, cross-kingdom interaction, enterocytes

## Abstract

The human gut acts as the main reservoir of microbes and a relevant source of life-threatening infections, especially in immunocompromised patients. There, the opportunistic fungal pathogen *Candida albicans* adapts to the host environment and additionally interacts with residing bacteria. We investigated fungal-bacterial interactions by coinfecting enterocytes with the yeast *Candida albicans* and the Gram-negative bacterium *Proteus mirabilis* resulting in enhanced host cell damage. This synergistic effect was conserved across different *P. mirabilis* isolates and occurred also with non-*albicans Candida* species and *C. albicans* mutants defective in filamentation or candidalysin production. Using bacterial deletion mutants, we identified the *P. mirabilis* hemolysin HpmA to be the key effector for host cell destruction. Spatially separated coinfections demonstrated that synergism between *Candida* and *Proteus* is induced by contact, but also by soluble factors. Specifically, we identified *Candida*-mediated glucose consumption and farnesol production as potential triggers for *Proteus* virulence. In summary, our study demonstrates that coinfection of enterocytes with *C. albicans* and *P. mirabilis* can result in increased host cell damage which is mediated by bacterial virulence factors as a result of fungal niche modification *via* nutrient consumption and production of soluble factors. This supports the notion that certain fungal-bacterial combinations have the potential to result in enhanced virulence in niches such as the gut and might therefore promote translocation and dissemination.

## 1 Introduction

The human body is densely colonized by high numbers of bacteria and fungi. They reside in different niches, which vary regarding temperature, moisture, surface structure, oxygen and nutrient availability, presence of other microbes, and interaction with the human immune system (Ribet and Cossart, 2015; Kapitan et al., 2019; Kruger et al., 2019). The gut is the most densely colonized anatomical niche, and the associated microbiota contributes to health and disease (Dekaboruah et al., 2020). Residing microbes facilitate food degradation, vitamin synthesis, and training of immunotolerance towards harmless colonizers (Dekaboruah et al., 2020). Yet, the gut is also one of the main reservoirs of opportunistic pathogens (Gouba and Drancourt, 2015). Chronic inflammation as well as antibiotic treatment can lead to reduced microbial diversity in the gut resulting in increased numbers of facultative pathogens (Zaborin et al., 2014; Gong et al., 2016; Armstrong et al., 2018; Zheng et al., 2020). This situation promotes translocation of gut microbes from the lumen into the blood stream followed by systemic dissemination (Gouba and Drancourt, 2015; Dekaboruah et al., 2020).

Prolonged, broad-spectrum antibiotic treatment is also one of the main risk factors for disseminated candidiasis (Miranda et al., 2009; Gouba and Drancourt, 2015; Ribet and Cossart, 2015; Lagunes and Rello, 2016; Iacob and Iacob, 2019; Shukla and Sobel, 2019; Mora Carpio and Climaco, 2021), a condition still associated with mortality rates of 30–40% despite antifungal treatment (Koehler et al., 2019). The most common cause of candidiasis is the yeast *Candida albicans* (Turner and Butler, 2014; Zhai et al., 2020) which is also a commensal colonizer on mucosal surfaces of 70–80% of the human population (Eggimann et al., 2015; Niemiec et al., 2017). Most blood stream infections with *C. albicans* are considered endogenous, originating from within the patient, and recent evidence shows that strains causing systemic candidiasis originate from the gut (Miranda et al., 2009). Within the gut, *C. albicans* interacts not only with the host, but also bacteria of the microbiota (Mirhakkak et al., 2021). Antagonistic interactions have been described, for example with *Bacteroides thetaiotaomicron*, which confers colonization resistance (Fan et al., 2015). While abundance of *B. thetaiotaomicron* and other benign commensals is often reduced by antibiotic therapy, the resulting low-diversity microbiota can become dominated by opportunistic bacterial pathogens, such as members of the *Enterobacteriacae*, and *C. albicans* (Zaborin et al., 2014). Synergistic interactions between the fungus and such bacteria might affect the likelihood of translocation across the gut barrier and subsequent disseminated infection, but have not been studied in much detail (Peleg et al., 2010; Kruger et al., 2019; Zhai et al., 2020; Santus et al., 2021).

Similarly to *C. albicans*, the Gram-negative bacterium *P. mirabilis* is a known commensal of the human gut (Hamilton et al., 2018). Due to its very low abundance (< 0.05%) in healthy gut microbiota and consequently undersampling in stool and failed detection by 16S profiling, its exact prevalence in healthy individuals is less well determined than for other *Enterobacteriaceae* – it is estimated to be around 3% or higher (Muller, 1986; Hamilton et al., 2018). However, *P. mirabilis* has been described as part of the dysbiotic gut microbiota in ulcerative colitis, Crohn’s disease, and rheumatic arthritis (Kanareykina et al., 1987; Elias-Oliveira et al., 2020; Zhang et al., 2021). Furthermore, inherent resistance to several antibiotics, such as polymyxins, tigecycline, and tetracycline, results in increased *P. mirabilis* abundance upon antibiotic treatment. *P. mirabilis* is known to spread from the gut to other niches of the human body, most prominently the urinary tract where it causes urinary tract infections, subsequent bladder and kidney stone formation, ascending pyelonephritits, or urosepsis (Armbruster and Mobley, 2012; Armbruster et al., 2018). In catheter-associated urinary tract infections *P. mirabilis* is frequently accompanied by other bacteria, such as *Escherichia coli*, *Morganella morganii*, *Enterococcu*s spp., or *Providencia stuartii* (Warren et al., 1982; Jacobsen and Shirtliff, 2011; Brauer et al., 2019). Therefore, its interactions with other bacteria have been studied in this context: Alteri *et al.* showed that *P. mirabilis* and *E. coli* cause urinary tract infections (UTIs) efficiently and more persistent when combined in a respective mouse model (Alteri et al., 2015). Similarly, studies by Armbruster et al. demonstrated that *P. mirabilis* pathogenicity increases during coinfection in a catheter-associated UTI (CAUTI) and bladder infection model in mice when combined with *Providencia stuartii* (Armbruster et al., 2017a; Armbruster et al., 2017b). During polymicrobial biofilm formation of *P. mirabilis* with *Klebsiella pneumoniae*, *E. coli*, or *M. morganii* in artificial urine, *Proteus*-mediated alteration of the medium led to reduced viability of all other microbes (Juarez et al., 2020). However, the interaction of *P. mirabilis* with other microbes in the context of the gut and its role as a member of the gut microbiota has not yet been addressed in much detail (Seo et al., 2015; Hamilton et al., 2018).

In the present study, we investigated the interaction of *C. albicans* and *P. mirabilis in vitro* using an enterocyte infection model. Coinfection resulted in significantly increased host cell damage. This synergistic effect was dependent on the *P. mirabilis* hemolysin HpmA but independent of *C. albicans* Als3-mediated adhesion, filamentation, or candidalysin. Both physical contact and multifactorial niche modification by *C. albicans* promoted host cell damage by the bacterium. This implies the potential of cross-kingdom microbial interactions to alter microbial virulence and identifies *C. albicans* and *P. mirabilis* as a potentially detrimental combination in certain niches of the human body.

## 2 Materials and Methods

### 2.1 Cultivation of Fungal and Bacterial Strains

A list of all strains used in this study is provided in **Supplementary Table 1**. All strains were kept as frozen stock with 25% (V/V) glycerol at -80°C. From the stocks, fungal strains were streaked on YPD (20 g/l peptone, BD; 10 g/l yeast extract, Roth; 20 g/l glucose, Roth) agar plates and incubated for 24 h at 30°C. Unless stated otherwise, a single colony was then transferred into liquid YPD and incubated overnight (16–18 h) at 30°C with vigorous shaking. Fungal cells were harvested by centrifugation at 10,000 × g for 1 min, washed twice with an equal volume of phosphate-buffered saline (PBS) and resuspended in PBS. Cell numbers were determined microscopically using a Neubauer chamber and cell density was adjusted to the desired concentration with PBS. For heat-inactivation, the adjusted cell suspension was incubated at 65°C for 1 h (Hosseinzadeh and Urban, 2013).

Bacterial strains were streaked from stocks onto Todd-Hewitt-Bouillon (THB, #X936, Carl Roth) agar plates and incubated for 24 h at 37°C. A single colony (alternatively ≈ 1 µl portion for swarming bacteria) was transferred into liquid THB and incubated overnight at 37°C with vigorous shaking. From the resulting stationary culture, a subculture was prepared by adjusting the OD_600_ to 0.1 in fresh THB and incubating at 37°C with vigorous shaking to an OD_600_ ≈ 1 (exponential phase). If necessary, THB was supplemented with appropriate antibiotics (**Supplementary Table 1**). Bacteria were harvested by centrifugation at 13,000 × g for 1 min, washed twice with PBS and resuspended in PBS. The OD_600_ was measured and cell density was adjusted to the desired concentration with PBS based on OD_600_-CFU-correlations (**Supplementary Table 1**). Infection suspensions were prepared in PBS.

### 2.2 Enterocyte Model

Brush border-forming C2BBe1 (CRL-2102™, ATCC; RRID : CVCL_1096) and mucus-secreting HT29-MTX (HT29-MTX-E12, Sigma; RRID : CVCL_G356) cells were cultured and maintained in DMEM (high glucose DMEM, #41965, Gibco) supplemented with 10% (V/V) FCS, 0.01 mg/ml holo-transferrin (#616397, Merck) and 1% V/V non-essential amino acids (#11140, Gibco) under standard cell culture conditions: 37°C, 5% CO_2_, ambient oxygen, humidified atmosphere, static incubation. For infection experiments, C2BBe1 and HT29-MTX cells were harvested by accutase (#ACC-1B, Capricorn) or trypsin (#25300, Gibco) digest, respectively. After determining the number of viable cells using a Neubauer chamber and trypan blue staining, cells were mixed in a 70/30 ratio to 10^5^ cells/ml in supplemented DMEM and seeded into polystyrene culture vessels (multiwell plates) treated with 10 µg/ml collagen I (#A10483-01, Thermo Fisher). The following volumes were used per well: 200 µl in 96-well, 1 ml in 24-well, and 5 ml in 6-well format. Cells were matured for 14 days with regular medium exchange. Before infection, the medium was removed and replaced with KBM™ Gold (#00192151, Lonza) medium after washing the cells with PBS.

### 2.3 Infection of Enterocytes, Quantification of Enterocyte Damage and Microbial Burden

For infection, mixed C2BBe1/HT29-MTX cultures were prepared as described above. Infection experiments were performed in KBM medium due to its high pH buffering capacities. The fungal multiplicity of infection (MOI) was calculated in relation to the total enterocyte number initially seeded into each well. For infection, 10µl fungal suspension adjusted to the desired concentration, or PBS for control, were added to each well. Unless indicated otherwise, fungi were used at MOI 10. After addition of fungal cells, enterocytes were incubated for 24 h under standard cell culture conditions. Then bacterial cells were added (10 µl) at MOI 1 unless indicated otherwise, followed by further incubation for 5 h. The supernatant was removed and used to determine host cell damage by quantification of lactate dehydrogenase release using the colorimetric cytotoxicity detect kit (#11644793001, Sigma-Aldrich) according to the manufacturer’s guidelines. Absorption at 485 nm (A_485_) was corrected by subtracting A_660_. Enterocytes lyzed with 0.25% (V/V) Triton-X 100 served as high control, untreated/uninfected enterocytes as low control. Relative host cell damage was calculated as % Triton-induced LDH release as follows: $\frac{A_{infection}-A_{low\;control}}{A_{high\;control}-A_{low\;control}}\times 100$ . For comparison of mono- and coinfections, the fold damage coinfection (FDC) was determined as follows: $\frac{\%\;damage\;coinfection}{\left(\%\;damage\;fungus\;+\;\%\;damage\;bacterium\right)}$ .

For the quantification of bacterial and fungal burden, the supernatant was not removed, but well contents were scratched, vigorously resuspended, and transferred into a fresh reaction tube. To disrupt fungal clumps, 1 mg/ml zymolase (Amsbio, #120491-1) was added and tubes were incubated for 1 h at 37°C with 500 rpm shaking *prior* to serial dilution and plating on agar plates. Selective plates were used to differentiate bacteria and fungi: For *P. mirabilis*, CLED (#2835, Carl Roth) agar plates were supplemented with 50 µg/ml nystatin to suppress fungal growth; for *C. albicans*, YPD plates were supplemented with 50 µg/ml gentamycin and 100 µg/ml doxycycline to suppress bacterial growth. pH of cell culture media was determined using a microelectrode (Orion™ PerpHecT™ ROSS™; Thermo Fisher Scientific).

#### 2.3.1 Cell Contact Assay

If spatial separation of microbes and/or enterocytes was required during infection, host cells were seeded into a 24-well plate and maturated as described above. Infection suspensions were prepared as described above, but inoculated either into the well or into a hanging PET cell culture insert with pore size 0.4 µm (Merck, # MCHT24H48). Incubation and host cell damage quantification were performed as described above.

### 2.4 Preparation of *Candida*-Conditioned Medium

To analyze the role of soluble factors released or depleted by *C. albicans* during infection, *Candida*-conditioned medium (CCM) was prepared. For this, *C. albicans* cultures were prepared as described above. Then, 10^6^ C*. albicans* cells/ml were inoculated into KBM in suitable multiwell plates and incubated under standard cell culture conditions for 24 h. The supernatant was harvested and sterile-filtered using a pore size of 0.2 µm. Aliquots were frozen at -20°C and thawed on demand in a water bath at 37°C *prior* to experiments. For size exclusion treatment, aliquots were thawed on ice and CCM was separated through centrifugal filters with molecular weight cut offs between 3 and 100 kDa (Amicon^®^Ultra, Millipore). For heat-inactivation CCM was incubated for 30 min at 72°C before the experiment. KBM treated in the same way was used as control. The pH of CCM and KBM was measured using a pH electrode (Orion™ PerpHecT™ ROSS™; Thermo Fisher Scientific) after equilibration to cell culture conditions for 5 h.

### 2.5 Metabolite Quantification in KBM and CCM

The commercially available kits GLUC3 and UREAL (cobas), respectively, were used to determine the concentration of glucose and urea in supernatants after sterile-filtering using a pore size of 0.2 µm. The detection limits reported by the manufacturer are 0.11 mM and 0.5 mM for glucose and urea, respectively.

### 2.6 Farnesol Quantification and Effects of Farnesol on Bacteria

To determine the concentration of farnesol produced by *C. albicans*, fungal cells were incubated in suitable multiwell plates under cell culture conditions. After 24 h, supernatants and cell pellets were generated by centrifugation for 15 min at 4000 × g at 4°C. Each type of samples was extracted by incubation with 10 ml pentane containing 0.2 ng/µl nonylacetate as internal standard for 3 h at 8°C with vigorous shaking. KBM spiked with 0.2% 1 M (*E*,*E*)-farnesol (0.2 mM) was included as additional control. The organic phase was collected and concentrated under nitrogen flow to a volume of approximately 200 µl. Qualitative and quantitative analysis of pentane extracts was conducted using an Agilent 6890 Series gas chromatograph coupled to an Agilent 5973 quadrupole mass selective detector (Agilent Technologies, Santa Clara, CA, USA; interface temp, 250°C; quadrupole temp, 150°C; source temp, 230°C; electron energy, 70 eV) or a flame ionization detector (FID) operated at 300°C, respectively. The constituents of the pentane extracts were separated using a ZB5 column (Phenomenex, Aschaffenburg, Germany; 30 m × 0.25 mm × 0.25 µm) and He (MS) or H_2_ (FID) as carrier gas. The sample (1 µL) was injected without split at an initial oven temperature of 80°C. The temperature was held for 2 min and then increased to 240°C with a gradient of 12°C min^−1^, and then further increased to 320°C with a gradient of 100°C min^−1^ and a hold of 2 min. *(E,E)-*farnesol was identified by comparison of its retention time and mass spectrum to those of an authentic standard obtained from Sigma (#F203).

To test for bactericidal effects of farnesol towards *P. mirabilis*, bacteria were inoculated into KBM at 10^5^ CFUs/ml, 10 mM farnesol dissolved in MeOH (final 1% (V/V)) was added, and cells were incubated for 5 h at 37°C with vigorous shaking. MeOH (final 1% (V/V)) was used as control. After incubation, samples were serially diluted and plated on CLED agar for CFU quantification.

### 2.7 Gene Expression Analysis

#### 2.7.1 Nucleic Acid Isolation

For gene expression analysis, RNA of *Proteus mirabilis* HI4320 was isolated using the Universal RNA Purification Kit (Roboklon). Similarly to the proteomics analysis, *P. mirabilis* was inoculated at 10^5^ CFUs/ml into KBM or CCM and incubated for 5 h under static cell culture conditions. Infections were performed in 6-well plates with 30 ml per condition. After incubation, all steps were performed at 4°C or on ice. Bacteria were detached by scraping them off the plastic and the resulting bacteria suspension was transferred into a 50 ml Falcon tube. Bacteria were harvested by centrifugation at 3,500 × g for 10 min. The supernatant was discarded, and the pellet resuspended in 500 μl RL buffer with 10%V/V mercaptoethanol freshly added (RL buffer as part of Roboklon RNA purification Kit, see 2.2.1). The slurry was transferred into a screwcap reaction tube filled with 500 μl glass mill beads (Mini-BeadBeater Glass Mill Beads 0.5 mm, Biospec Products). To lyze the cells, reaction tubes were shaken vigorously in a bead beater (Precellys™ Control Device; Bertin technologies) with 6,500 rpm for 20s, cooled one ice for 15 sec, and shaken again. Beads and cell debris were sedimented by centrifugation for 5 min at 10,000 × g. The remaining lysate was then transferred onto the homogenization column and the isolation protocol was executed according to the manufacturer’s protocol. After elution in RNase-free water, purity and quantity were determined using a NanoDrop spectrophotometer (Peqlab Nanodrop 2000). RNA was either used directly or kept at -20°C for short term storage. To ensure sufficient RNA quality, samples were examined using the 2100 Bioanalyzer system by Agilent. RNA samples were prepared and applied following the manufacture’s guidelines.

#### 2.7.2 Reverse Transcription of RNA into cDNA

Prior to conversion to cDNA, possible DNA contaminations in the RNA samples were digested using TURBO DNA-free Kit*™* (Thermo Fischer). To transcribe RNA into cDNA, the SuperScript™ III/VI First-Strand Synthesis System (Thermo Fisher) was used according to manufacturer guidelines. A total of 250 ng RNA was used in the reaction. The cDNA was then diluted 1:4 with nuclease-free water for further use in quantitative polymerase chain reaction (qPCR).

#### 2.7.3 Primer Design and Verification

To determine if the hemolysin gene *hpmA* is differentially regulated during coinfection, qPCR was conducted. Two primers were designed using NCBI genome database and IDT primerQuest Tool R (Supp. Tab. 2). In addition, primers for three different reference genes were included. Primers were purchased from Biomers. For all primers, primer efficiency was determined using genomic DNA of a *P. mirabilis* HI4320 overnight culture extracted using the DNeasy Blood and Tissue Kit (Qiagen) following the instructions for bacterial cultures. DNA was stored at -20°C, thawed on ice before use, and serially diluted for qPCR. Efficiency was tested using the Bio-Rad CFX96 Touch Real-Time PCR System and the GoTaqR qPCR Master Mix. Primers deemed suitable if they reached ≥ 70%.

#### 2.7.4 Reverse Transcriptase Quantitative Polymerase Chain Reaction

To set up a qPCR, we prepared a reaction mixture according to GoTaqR RT-PCR Kit (Promega) guidelines; the final concentration of the primers was 0.1 pmol/µl. The Real Time PCR system Bio-Rad CFX96 Touch Real-Time PCR System was used to perform the PCR. No-template controls were used as negative controls. The cycle was as follows: activation 95°C, 10 min; denaturation 95°C 15 sec; annealing 58°C 20 sec; elongation 72°C 20 sec (back to denaturation for 44 cycles); denaturation 95°C 15 sec, melt curve from 65°C to 95°C for 15 s (0.5°C). In order to determine relative gene expression, the Δ/ΔCt values were used to calculate the fold change (FC) after normalization to a reference gene (Livak and Schmittgen, 2001). We used three different reference genes: *rpoA* (Pearson et al., 2011), *secB* and *cpxA* (Wang et al., 2010). A FC > 1 implies upregulation.

### 2.8 Protein Expression Analysis

#### 2.8.1 Proteomics Sample Preparation

In order to investigate the extent to which HpmA protein expression differs in *P. mirabilis* upon incubation in KBM versus CCM, quantitative proteomics was performed. For this purpose, 200 ml of KBM or CCM per condition were divided into four 175 cm² cell culture flasks (Sarstedt), inoculated with the normal *in vitro* model infection dose of 10^5^ *Proteus* CFUs/ml and incubated under static cell culture conditions. After incubation, samples were immediately cooled on ice and bacteria were harvested using a cell scraper, vigorous pipetting and centrifugation for 15 min at 4°C and 4,200 × *g*. The supernatants were collected separately in acid washed plastic Erlenmeyer flasks, one tablet of cOmplete Ultra Protease Inhibitor cocktail (Roche) per 50 ml was added, and the pH was set to 2.0 with trifluoroacetic acid (TFA). Then they were sterile-filtered using a 0.2 µm vacuum filter system with PES membrane (VWR/TPP) to remove bacteria and other particles and stored at -80°C. The pellets were resuspended in 1.5 ml of ice-cold PBS and centrifuged at 13,000 × *g* for one min. This was followed by a second washing step with ice-cold distilled water. The washed pellets were taken up in 1 ml lysis buffer (aqueous solution of 1% SDS, 150 mM NaCl, 100 mM triethylammonium bicarbonate [TEAB] and one tablet cOmplete Ultra protease inhibitor cocktail per 10 ml) and transferred to 1.5 ml screw cap tubes filled with approximately 500 μl glass beads and disrupted in the BeadBeater^®^ for 40 seconds at 4 m/s² (see RNA extraction). The mixture was centrifuged at 10,000 × *g* for three minutes at 4°C and the lysate was separated from the beads. Nucleic acids were digested with 400 U Benzonase (Novagen) followed by 15 min centrifugation at 18,000 × *g* (4°C).

The proteins of the supernatant (secretome) were concentrated by solid phase extraction (SPE) on a Chromabond™ C4 SPE column (Macherey-Nagel™), washed with 0.1% TFA and eluted in 4 ml of 0.1% TFA in 80:20 (v/v) acetonitrile (ACN)/water. The eluate was evaporated in a vacuum concentrator (Eppendorf) to almost dryness. Eluates were either evaporated or precipitated by 20% trichloroacetic acid (TCA) for 30 min on ice with a subsequent washing step using 90% acetone (centrifugation at 20,000 × *g* for 15 min at 4°C). Samples were resolubilized in 150 µl 50 mM TEAB in 50:50 (v/v) water/2,2,2-trifluoroethanol (TFE). Protein concentrations were measured by the Merck-Millipore Direct Detect system.

Proteome and secretome samples were reduced and alkylated by 10 mM Tris(2-carboxyethyl)phosphine and 12.5 mM 2-chloroacetamide (final concentrations) in the dark for 30 min at 70°C and 500 rpm shaking. For further purification, proteins were precipitated with 9 times the volume of cold acetone at -80°C overnight. Samples were centrifuged for 15 min at 20,000 × *g* at 1°C. The resulting pellets were washed twice with 1 ml of 90% acetone at -80°C and then air-dried. Proteins were resuspended in 200 μl of TEAB buffer and the protein concentration was measured as described above. Proteolytic digestion was performed by incubation with trypsin/LysC mixture (Promega) at a protein/protease ratio of 25:1 for 18 hours. Samples were filtered with a modified PES 10 kDa MWCO spin filters (VWR) at 14,000 × *g* for 15 min. Further purification was performed by water-saturated ethyl acetate extraction using the protocol of Yeung and Stanley (Yeung and Stanley, 2010). Finally, all samples were evaporated to dryness in a vacuum concentrator, resolubilized in 30 µl of 0.05% TFA and 2% ACN, filtered through 0.2 µm Merk-Millipore Ultrafree-MC hydrophilic PTFE spin filters (14,000 × *g* for 15 min), and transferred to HPLC vials.

#### 2.8.2 LC-MS/MS-Based Proteome Analysis

Each sample was measured in three analytical replicates. LC-MS/MS analysis was performed on an Ultimate 3000 RSLCnano system connected to either (#1) a QExactive Plus or (#2) a QExactive HF mass spectrometer (both Thermo Fisher Scientific, Waltham, MA, USA). Peptide trapping for 5 min on an Acclaim Pep Map 100 column (2 cm x 75 µm, 3 µm) at 5 µL/min was followed by separation on an analytical Acclaim Pep Map RSLC nano column (50 cm x 75 µm, 2µm). Mobile phase gradient elution of eluent A (0.1% (v/v) formic acid in water) mixed with eluent B (0.1% (v/v) formic acid in 90/10 acetonitrile/water) was performed using the following gradient: 0–4 min at 4% B, 10 min at 7% B, 50 min at 12% B, 100 min at 16% B, 150 min at 25% B, 175 min at 35% B, 200 min at 60%B, 210–215 min at 96% B, 215.1–240 min at 4% B.

Positively charged ions were generated at spray voltage of 2.2 kV using a stainless-steel emitter attached to the Nanospray Flex Ion Source (Thermo Fisher Scientific). Data acquisition was in Full MS/data-dependent MS2 (#1) Top10 or (#2) Top 15 mode. Precursor ions were monitored at m/z 300–1500 at a resolution of (#1) 140,000 or (#2) 120,000 full width at half maximum (FWHM) using a maximum injection time (ITmax) of 120 ms and an AGC (automatic gain control) target of 3 × 10^6^. Precursor ions with a charge state of z=2-5 were filtered at an isolation width of *m/z* 1.6 amu for further HCD fragmentation at 30% normalized collision energy (NCE). MS2 ions were scanned at (#1) 17,500 or (#2) 15,000 FWHM (ITmax=100 ms, AGC= 2 × 10^5^) using a fixed first mass of *m/z* 120 amu. Dynamic exclusion of precursor ions was set to 30 s and the minimum AGC target for Precursor ions selected for HCD fragmentation was set to 1e3. The LC-MS/MS instruments were controlled by Chromeleon 7.2 and Tune 2.8.

#### 2.8.3 Protein Database Search

Tandem mass spectra were searched against the UniProt database (2021/04/22) *Candida albicans* SC5314 (https://www.uniprot.org/proteomes/UP000000559) and *Proteus mirabilis* HI4320 (https://www.uniprot.org/proteomes/UP000008319) using Proteome Discoverer (PD) 2.4 (Thermo) and the algorithms of Mascot 2.4.1 (Matrix Science, UK), Sequest HT (version of PD2.4), MS Amanda 2.0, and MS Fragger 3.2. Two missed cleavages were allowed for the tryptic digestion. The precursor mass tolerance was set to 10 ppm and the fragment mass tolerance was set to 0.02 Da. Modifications were defined as dynamic Met oxidation, protein N-term acetylation and/or loss of methionine, as well as static Cys carbamidomethylation. A strict false discovery rate (FDR) < 1% (peptide and protein level) and a search engine score of >30 (Mascot), > 4 (Sequest HT), >300 (MS Amanda) or >8 (MS Fragger) were required for positive protein hits. The Percolator node of PD2.4 and a reverse decoy database was used for qvalue validation of spectral matches. Only rank 1 proteins and peptides of the top scored proteins were counted. Label-free protein quantification was based on the Minora algorithm of PD2.4 using the precursor abundance based on intensity and a signal-to-noise ratio >5. Normalization of the proteome samples was performed by using the total peptide amount method. Imputation of missing quan values was applied by using abundance values of 75% of the lowest abundance identified per sample. Differential protein abundance was defined as a fold change of >4, ratio-adjusted p-value <0.05 (p-value/log4ratio) and at least identified in 2 of 3 replicates. The mass spectrometry proteomics data have been deposited to the ProteomeXchange Consortium *via* the PRIDE (Perez-Riverol et al., 2019) partner repository with the dataset identifier PXD031222.

### 2.9 Scanning Electron Microscopy

For scanning electron microscopy (SEM) analysis, sterile glass slides were placed into 24-well plates and treated with collagen as described for cell culture. Enterocyte culture and infection were performed as described above; additionally, Bacteria/fungi were added on slides without enterocytes. After incubation, the medium was replaced with cacodylate buffer for 10 min (0.1 M, pH 7.2), followed by replacement with cacodylate buffer containing freshly added glutaraldehyde (2.5% (V/V)). Cells were fixed over night at 4°C, before washing three times with cacodylate buffer. After dehydration with EtOH in increasing concentrations (30%, 50%, 70%, 80%, 90%, 100%, 100%; 15 min each), samples were critical point dried in a Leica EM CPD300 Automated Critical Point Dryer and then sputter-coated with 30 nm gold using a BAL-TEC SCD005 Sputter Coater. Samples were analyzed at different magnifications with a Zeiss (LEO) 1530 Gemini field emission scanning electron microscope (Carl Zeiss GmbH, Oberkochen, Germany) at 7kV acceleration voltage and a working distance of 5 mm using an InLens secondary electron detector.

### 2.10 Statistical Analyses

GraphPad Prism 9 was used for data analyses. The number of biological replicates is indicated in the legend of each graph. Data is represented as arithmetic mean ± SD unless indicated otherwise in the figure legend, and was tested for normality distribution using the Shapiro-Wilk test and D’Agostino-Pearson (if n was large enough) normality test. Based on this, parametric tests were used. Depending on the data set, two-sided unpaired t-test, One-Way ANOVA with Dunnett’s multiple comparison test, 2-way ANOVA and Šídák’s multiple comparisons test or Tukey’s multiple comparisons test were used as indicated in the figure legends. P values ≤ 0.05 were considered significant.

## 3 Results

### 3.1 Coinfection With *C. albicans* and *P. mirabilis* Significantly Increases Enterocyte Damage

**Figure 1:**
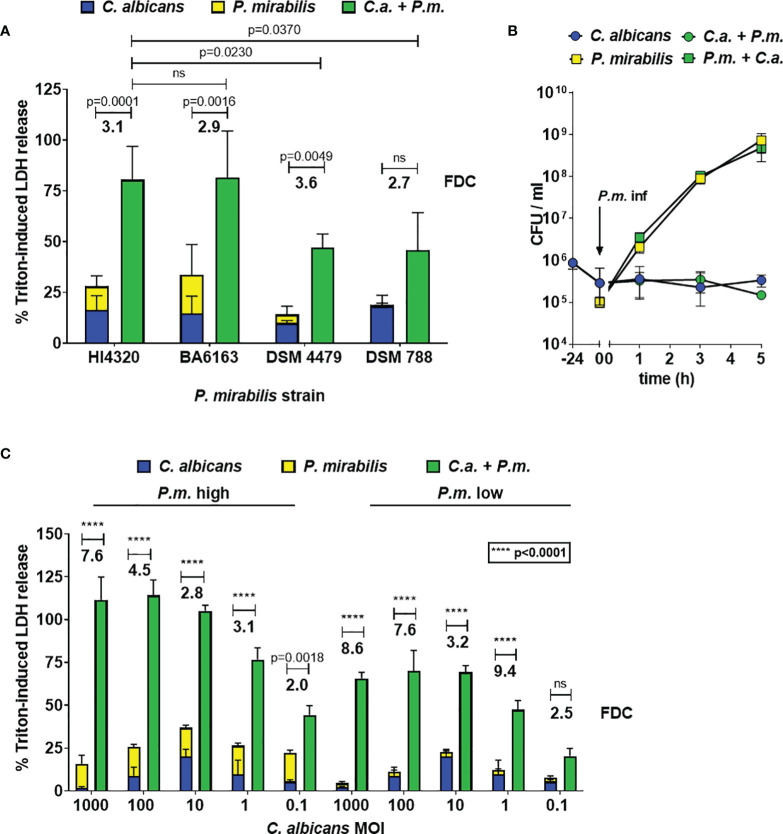
Synergistic enterocyte damage caused by *C. albicans* – *P. mirabilis* coinfection. Enterocytes were infected for 24 h with *C. albicans* SC5314 at MOI 10 and subsequently with different *P. mirabilis* wildtype strains at MOI 1 for 5 h **(A)** Host cell damage was assessed by LDH release related to a Triton-induced high control corrected for spontaneous cell death of uninfected enterocytes. Means and SD from 3-5 independent experiments are plotted; the sum of monoinfection damage (*C. albicans*: blue, *P. mirabilis*: yellow; shown as stacked) was compared to coinfection damage (green) by Two-way ANOVA and Šídák’s multiple comparisons test, and the increase relative to the sum of monoinfections (mean fold damage coinfection, FDC) is indicated for each *P. mirabilis* strain. Coinfection damage with *P. mirabilis* HI4320 was compared with coinfection damage of other *P. mirabilis* strains by One-Way ANOVA with Dunnett’s multiple comparison test. Significant differences are indicated by absolute p values in the graph, ns: not significant. **(B)** To determine microbial burden during infection, enterocytes were infected for 24 h with *C. albicans* SC5314 at 10^6^ CFUs/ml (≙ MOI 10) and subsequently with *P. mirabilis* HI4320 at 10^5^ CFUs/ml (≙ MOI 1) for 5 h At time points indicated, well contents were harvested, treated with 1 mg/ml zymolyase, and plated for CFU determination. Mean and range from three independent experiments are plotted (*P. mirabilis*: square, *C. albicans*: circle). CFUs of each microbe in mono- and coinfection, respectively, were compared by Two-Way ANOVA and Šídák’s multiple comparisons test; no significant differences were observed at any time point. **(C)** Infections were performed and evaluated as described for **(A)**; infection doses ranged from MOI 1000 to 0.1 for *C. albicans* SC5314 and MOI 1 (high dose) or 0.1 (low dose) was used for *P. mirabilis* HI4320.

To determine the effects of *C. albicans* and *P. mirabilis* coinfection on enterocyte damage, host cells were infected with *C. albicans* at MOI 10 for 24 h, followed by addition of *P. mirabilis* (MOI 1) for 5 h. Host cell damage was quantified by measuring the release of LDH into the supernatant. To compare mono- and coinfections, and to account for dosage effects in coinfection, LDH release after monoinfection with either microbe was summed up and compared with the LDH release after coinfection. Four *P. mirabilis* strains were tested, and for three of these strains the coinfection resulted in significantly higher damage (**Figure 1A**). Additionally, we calculated the fold damage coinfection (FDC) as a measure of synergism induction by relating the coinfection damage to the sum of monoinfections and found that it ranged from 2.7 with *P. mirabilis* strain DSM788 to 3.6 with *P. mirabilis* strain DSM4479 (**Figure 1A**). Similar results were obtained with eight clinical isolates of *P. mirabilis* from blood and urine, with mean FDCs ranging from 1.4 to 4.4 (**Figure S1**). Based on its average FDC of 3.1, the well-characterized *P. mirabilis* strain HI4320 (FDC 3.1) was chosen as a representative wildtype strain for all further experiments (Pearson et al., 2008).

One possible explanation for the increased coinfection damage is growth promotion of one or both microbes. We therefore quantified fungal and bacterial CFUs in mono- and coinfection of *C. albicans* and *P. mirabilis*. CFUs of both microbes did not differ between mono- and coinfection, excluding enhanced proliferation as the underlying cause of synergistic host cell damage (**Figure 1B**). This experiment also revealed rapid proliferation of the bacteria in the co-infection setting; although bacteria were added at a lower MOI than *C. albicans*, the bacterial CFU were 10-fold higher than fungal numbers within 1 h of co-infection, with the final CFU of *P. mirabilis* being over 1000-fold higher than fungal CFU. Thus, higher abundance of bacteria, reflecting the physiological ratio of bacteria and fungi in the gut, developed within the model in a short period of time.

Next, we determined the impact of the ratio of fungal to bacterial cells on damage. The coinfection damage increased with increasing *C. albicans* MOIs of 0.1 to 10 (**Figure 1C**). Further increase of the *C. albicans* MOI did not lead to an increase of coinfection damage, but to a reduction of monoinfection damage (**Figure 1C**). This might be explained by saturation of the enterocyte surface with fungal cells, and dose-dependent induction of fungal quorum sensing which negatively impacts filamentation (Polke and Jacobsen, 2017; Polke et al., 2017; Polke et al., 2018).

### 3.2 Typical Mucosal *Candida albicans* Virulence Traits Are Dispensable for Synergism

**Figure 2:**
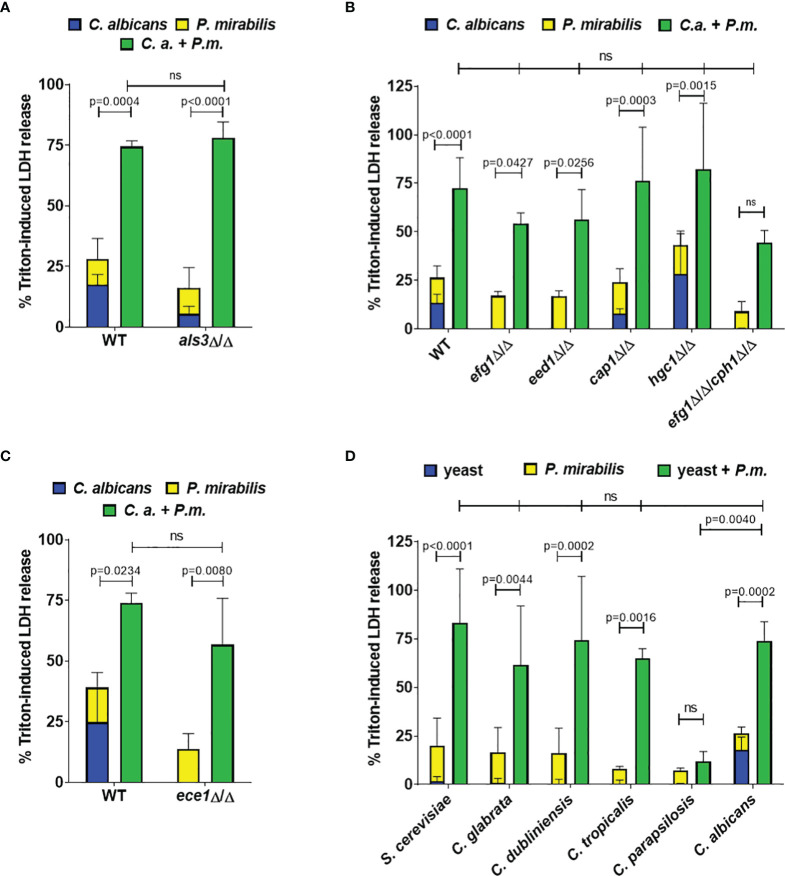
Synergistic damage does not depend on *C. albicans* filamentation and candidalysin, and is also induced by non-*albicans* yeast species. Enterocytes were infected for 24 h with the indicated fungal strains (MOI 10) and subsequently with *P. mirabilis* HI4320 (MOI 1) for 5 h Host cell damage was assessed by quantification of LDH and is shown relative to a Triton-induced high control. Means and SD from 3-4 independent experiments are plotted; summed-up monoinfections (*C. albicans*: blue, *P. mirabilis*: yellow; shown as stacked) were compared to coinfections (green) by Two-way ANOVA and Šídák’s multiple comparisons test; coinfection damage with *C. albicans* parental strains (WT) SC5314 or BWP17-CIp30 was compared with coinfection damage of corresponding mutants by two-sided unpaired t-test **(A, C)** or by One-Way ANOVA with Dunnett’s multiple comparison test **(B, D)**. Significant differences are indicated by absolute p values in the graphs, ns, not significant. **(A)** WT: *C. albicans* BWP17-CIp30, **(B)** WT: *C. albicans* SC5314; **(C)** WT: *C. albicans* BWP17-CIp30; **(D)** non-albicans *Candida* species and *S. cerevisiae*.

Increased enterocyte damage during coinfection might be caused by either fungal or bacterial virulence factors, or a combination of factors from both species. To determine the relative contribution of each microbe, we first performed coinfections using heat-killed *C. albicans* and found that inactivation of the fungus abolished the synergistic effect on damage except for the highest *Candida* MOI combined with high *Proteus* MOI (**Figure S2**). *C. albicans* filamentation is generally considered as the main virulence trait as it is accompanied by increased expression of adhesins and cytotoxic effectors (Sudbery, 2011). Therefore, we investigated the role of *C. albicans* adhesion, filamentation, and candidalysin production using homozygous deletion mutants. Deletion of the adhesin Als3 (*als3*Δ/Δ) slightly reduced enterocyte damage in *Candida* monoinfection but not coinfection (**Figure 2A**). Similarly, defects in filamentation had no significant impact on synergistically enhanced coinfection damage (*efg1*Δ/Δ, *eed1*Δ/Δ, *cap1*Δ/Δ, *hgc1*Δ/Δ), even for those strains that induced no detectable damage in monoinfection (*efg1*Δ/Δ, *eed1*Δ/Δ, *efg1*Δ/Δ*cph1*Δ/Δ; **Figure 2B**). Although *C. albicans hgc1*Δ/Δ coinfection damage was not significantly higher (p = 0.0586), it still exceeded the sum of monoinfections. This implies that *C. albicans* hypha-induced enterocyte damage is not required for the synergistic damage observed during coinfection. Consistent with this, deletion of the peptide toxin candidalysin (*ece1*Δ/Δ) did not reduce coinfection damage (**Figure 2C**). To determine if the induction of increased coinfection damage was specific to *C. albicans*, we performed coinfection experiments with *C. glabrata*, *C. dubliniensis*, *C. tropicalis*, *C. parapsilosis*, and *S. cerevisiae*. With the exception of *C. parapsilosis* significantly increased damage during coinfection was observed for all species, despite low cytotoxicity in monoinfections (**Figure 2D**). Thus, while increased coinfection damage depended on the presence of viable fungi, it was independent of the damage potential of theses fungi. This implies that synergistically enhanced damage is executed by bacterial virulence factors.

### 3.3 *P. mirabilis* Hemolysin Is Essential for Increased Coinfection Damage

*P. mirabilis* possesses a number of virulence factors, of which the cytotoxic hemolysin HpmA has been shown to mediate cell damage *in vitro* and to contribute significantly to virulence *in vivo* (Peerbooms et al., 1984; Peerbooms et al., 1985; Swihart and Welch, 1990a; b; Mobley et al., 1991; Alamuri et al., 2009; Seo et al., 2015). As recently reviewed by Armbruster et al., hemolysin contributes to bladder colonization during UTI and is primarily responsible for direct lysis of human renal proximal tubular epithelial cells (Armbruster et al., 2018). It is highly prevalent in clinical isolates from humans and animals (Swihart and Welch, 1990b; Mobley et al., 1991; Cestari et al., 2013; Sanches et al., 2019).

**Figure 3:**
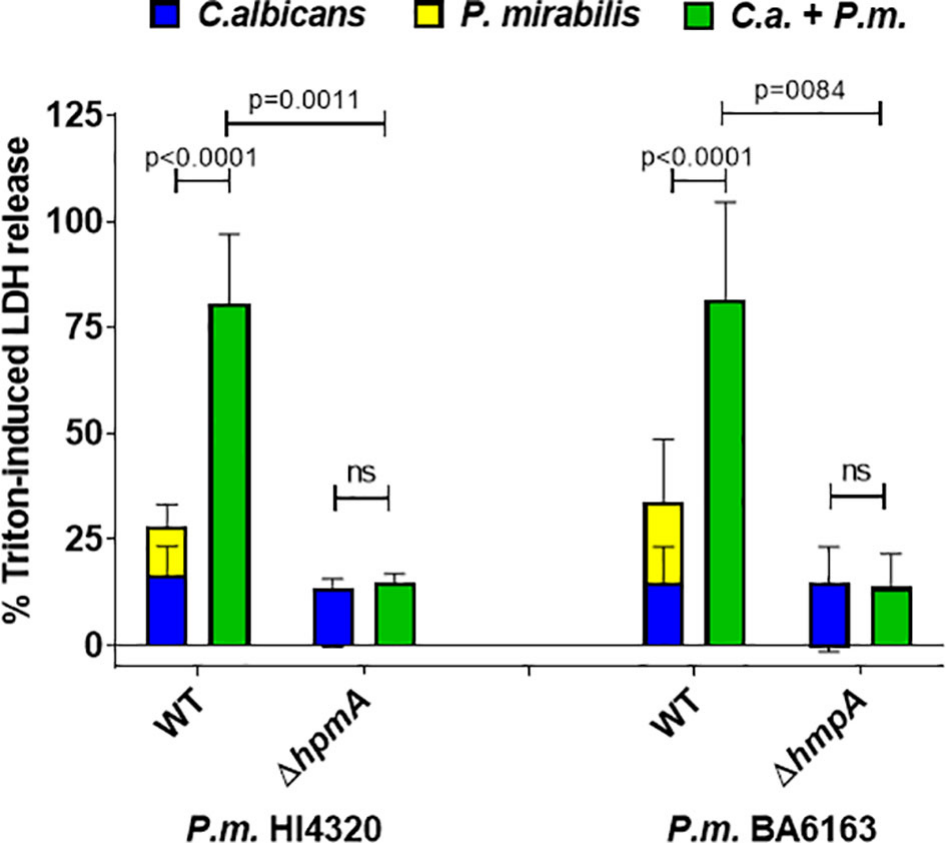
Hemolysin-deficient *P. mirabilis* strains are unable to execute synergistically enhanced coinfection damage. Enterocytes were infected for 24 h with *C. albicans* (*C.a.*) SC5314 at MOI 10 and subsequently with *P. mirabilis* (*P.m.*) HI4320 and BA6163 parental strains (WT) and corresponding hemolysin-deficient mutants at MOI 1 for 5 h. Host cell damage was assessed by LDH release related to a Triton-induced high control corrected for spontaneous cell death of uninfected enterocytes. Means and SD from 3-4 independent experiments are plotted; the sum of monoinfection damage (*C. albicans*: blue, *P. mirabilis*: yellow; shown as stacked) was compared to coinfection damage (green) by Two-way ANOVA and Šídák’s multiple comparisons test; coinfection damage with *P. mirabilis* WT and the corresponding mutant was also compared by unpaired two-sided t-test. Significant differences are indicated by absolute p values in the graph, ns, not significant.

We therefore hypothesized that this hemolysin might also play a significant role in our enterocyte infection model, and tested two hemolysin-deficient *P. mirabilis* mutants generated in different genetic backgrounds (Mobley et al., 1991; Seo et al., 2015). Both *hpmA* deletion mutants were unable to induce measurable cell damage in monoinfections (**Figure 3**). Furthermore, enterocyte damage after coinfection with these mutants was comparable to the damage induced by *C. albicans* alone, suggesting that the enhanced damage during coinfection with the corresponding wildtype strains is driven by the *P. mirabilis* hemolysin.

### 3.4 Both Physical Contact and Soluble Factors Contribute to *C. albicans-P. mirabilis* Synergism

**Figure 4:**
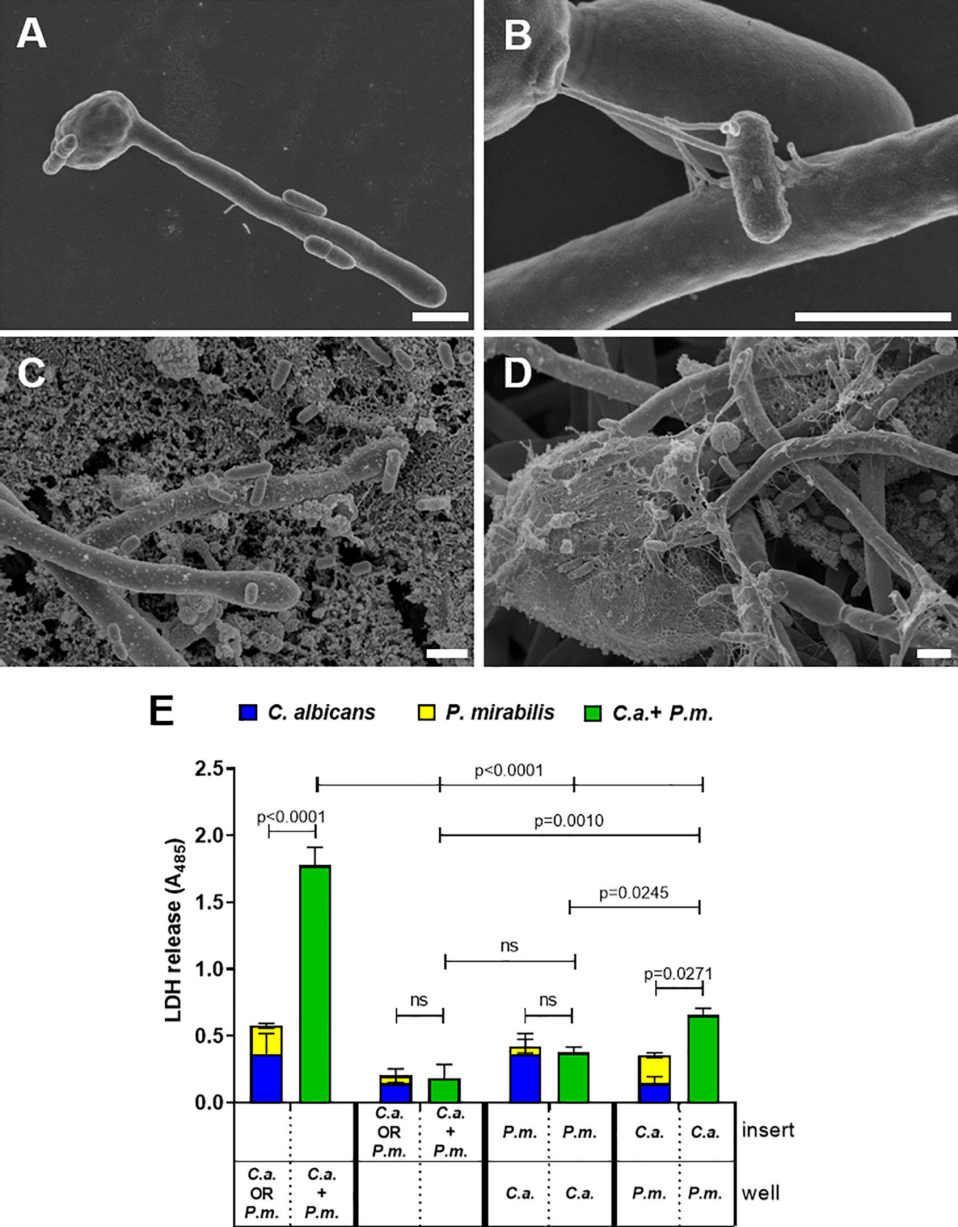
Physical interaction and soluble factors involved in synergistically enhanced enterocyte damage by *C. albicans* and *P. mirabilis.* Wells without (A+B) or with (C+D) enterocytes were inoculated for 24 h with *C. albicans* SC5314 at 10^5^ CFUs/ml and subsequently with *P. mirabilis* HI4320 at MOI 10^4^ CFUs/ml for 5 h Cells were fixed and analyzed by scanning electron microscopy (SEM). Magnifications are **(A)** 5000 ×, **(B)** 4000 ×, **(C)** 7000 x, and **(D)** 20,000 ×; scale bars represent 2 µm. Representative images of two independently performed experiments are shown. **(E)** Enterocytes were infected for 24 h with *C. albicans* (*C.a.*) SC5314 at MOI 10 and subsequently with *P. mirabilis* (*P.m.*) HI4320 at MOI 1 for 5 h Direct contact to host cells or among microbes was restricted by containing either bacteria or yeasts in a transwell insert with pore size 0.4 µm. Localization of respective microbes is depicted as “insert” or “well” (in contact with enterocytes). Host cell damage was assessed by LDH release. Means and SD from three independent experiments are plotted; the sum of monoinfection damage (*C. albicans*: blue, *P. mirabilis*: yellow; shown as stacked) was compared to coinfection damage (green) by Two-way ANOVA and Šídák’s multiple comparisons test. Coinfection damage of the different set ups were compared to each other by One-Way ANOVA with Tukey’s multiple comparison test Significant differences are indicated by absolute p values in the graph, ns, not significant.

In *P. mirabilis*, surface contact is associated with swarming induction and this in turn with increased expression of virulence effectors, including hemolysin (Allison et al., 1992b; Gygi et al., 1995; Liaw et al., 2000; Fraser et al., 2002; Jones et al., 2004; Belas and Suvanasuthi, 2005; Wang et al., 2006). Scanning electron microscopy (SEM) of coincubation and coinfection revealed adherence of bacterial cells to fungal yeast and hypha both in the absence (**Figures 4A, B**) and presence of enterocytes (**Figures 4C, D**). To determine if this physical contact is required for the increased damage during coinfection, we used transwell inserts to separate either *Proteus* or *Candida* alone or both microbes from enterocytes. Here, enterocyte damage caused by either *P. mirabilis* or *C. albicans* monoinfection was reduced if the microbes were physically separated from host cells (**Figure 4E**). This is consistent with the importance of hypha invasion for *C. albicans*-mediated damage (Gow et al., 2012). Similarly, coinfection damage was significantly reduced if both, *C. albicans* and *P. mirabilis*, were contained in the upper compartment compared to coinfection with mutual host cell contact (**Figure 4E**). With *C. albicans* on enterocytes, enterocyte damage was not influenced by the presence of *P. mirabilis* in the insert, whereas damage significantly increased with *P. mirabilis* on enterocytes if *C. albicans* was present in the upper compartment (**Figure 4E**). However, the resulting damage was significantly lower than that observed when both microbes were placed into the enterocyte compartment (**Figure 4E**), suggesting that physical interaction between the microbes is required for full synergism.

**Figure 5:**
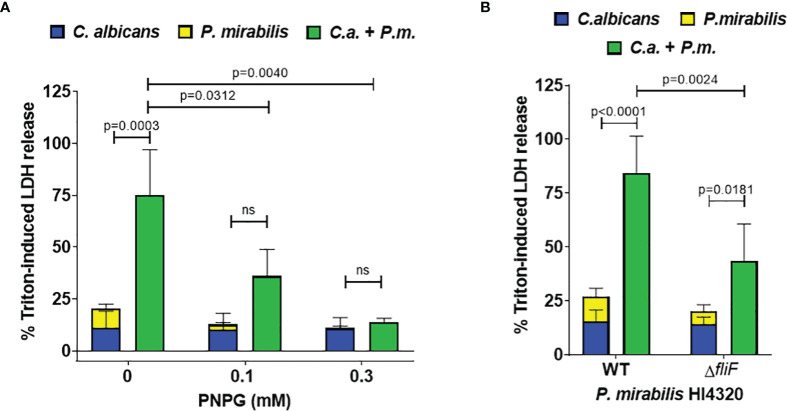
Synergistically enhanced coinfection damage is reduced by swarming interference. **(A)** Enterocytes were infected for 24 h with *C albicans* (*C.a.*) SC5314 at MOI 10 followed by *P. mirabilis* (*P.m.*) HI4320 in the absence or presence of either 0.1 or 0.3 mM p-nitrophenyl glycerin (PNPG) at MOI 1 for 5 h Host cell damage was assessed by LDH release related to a Triton-induced high control corrected for spontaneous cell death of uninfected enterocytes without PNPG. Means and SD from 4-5 independent experiments are plotted; the sum of monoinfection damage (*C. albicans*: blue, *P. mirabilis*: yellow; shown as stacked) was compared to coinfection damage (green) by Two-way ANOVA and Šídák’s multiple comparisons test. Coinfection damage of the different set ups were compared by One-Way ANOVA with Dunnett’s multiple comparisons test. Significant differences are indicated by absolute p values in the graph, ns, not significant. **(B)** Coinfections were performed and analyzed as described for **(A)** using a *P. mirabilis* mutant deficient in gene *fliF*. Means and SD from 5-7 independent experiments are plotted and analyzed as described for **(A)** for comparison of mono- and coinfection damage. Coinfection damage caused by WT and the *fliF* mutant was compared by unpaired two-sided t-test.

To analyze if contact-dependent synergistic damage by *P. mirabilis* involves swarming-associated gene regulation, mono- and coinfections were supplemented with the swarming inhibitor p-nitrophenyl glycerol (Williams, 1973; Liaw et al., 2000). Using 0.1 mM and 0.3 mM PNPG we observed a dose-dependent significant reduction of *P. mirabilis* monoinfection and coinfection damage (**Figure 5A**). The FDC was reduced from 3.9 in the untreated coinfection to 3.6 and 1.3 upon treatment with 0.1 and 0.3 mM PNPG, respectively. *C. albicans* monoinfection damage was unaffected. In addition, we performed coinfections using a swarming-impaired *P. mirabilis* mutant lacking the flagella gene *fliF* (Himpsl et al., 2008)(**Figure 5B**). Compared to coinfection with the *P. mirabilis* wildtype, the *fliF* mutant caused significantly less coinfection damage and a reduced FDC (3.2 *versus* 2.1). Taken together, these findings indicate that both physical interactions between the microbes and also soluble factors or alteration of medium composition by *C. albicans* contribute to the synergistically increased damage during coinfection.

### 3.5 *C. albicans* Secreted Factors and Medium Alteration Affect Host Cell Damage by *P. mirabilis*

**Figure 6:**
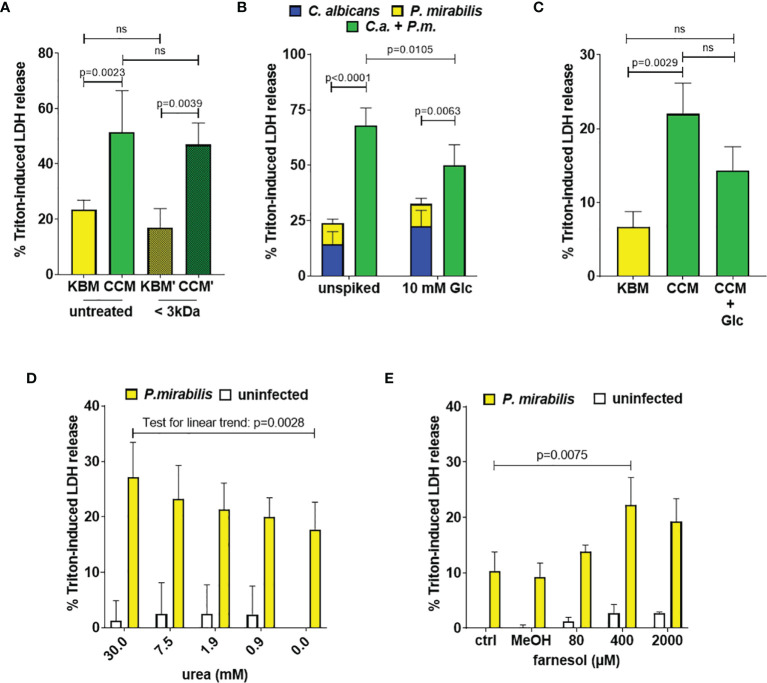
*Candida*-conditioned medium increases enterocyte damage caused by *P. mirabilis* infection. Enterocyte either previously infected with *C. albicans* SC5314 at indicated MOIs for 24 h or supplemented with CCM or KBM were infected with *P. mirabilis* HI4320 at MOI 1 for 5 h Host cell damage was assessed by LDH release relative to a Triton-induced high control corrected for spontaneous cell death of uninfected enterocytes. **(A)** *Candida*-conditioned medium (CCM) and a medium control (KBM) were treated by size-exclusion separation with a molecular weight cut-off of 3 kDa *prior* to the infection. Means and SD from 3 independent experiments using different batches of treated KBM (KBM’) and treated CCM (CCM’), respectively, are depicted. Host cell damage in untreated KBM or KMB’) and CCM or CCM’ were compared by One-Way ANOVA with Tukey’s multiple comparisons test. Significant differences are indicated by absolute p values in the graph, ns: not significant **(B)** After 24 h *C. albicans* infection, the medium was supplemented with 10 mM Glc just *prior* to infection with *P. mirabilis* HI4320 at MOI 1. Means and SD from 5 independent experiments are depicted. The sum of monoinfection damage (*C. albicans*: blue, *P. mirabilis*: yellow; shown as stacked) was compared to coinfection damage (green) by Two-way ANOVA and Šídák’s multiple comparisons test. Coinfection damage with and without glucose was compared using unpaired two-sided t-test. Significant differences are indicated by absolute p values in the graph, ns, not significant. **(C)** *P. mirabilis* moninfections in CCM were supplemented with 10 mM Glc and compared to untreated CCM or KBM. Means and SD from 3 independent experiments using independent preparations of CCM are depicted and were analyzed as described for **(A)**. **(D)** + **(E)** In KBM, *P. mirabilis* HI4320 at MOI 1 was supplemented with urea or farnesol in MeOH (final 1% V/V) at increasing concentrations. For farnesol, a 1% V/V MeOH control was included. Means and SD from 3 independent experiments are plotted. Data was analyzed by One-Way ANOVA with Dunnett’s multiple comparisons test to compare coinfection damage with urea to coinfection without urea **(D)** or coinfection with farnesol to the methanol infection control **(E)**; a test for linear trend was performed to determine if the dose had a significant impact on damage. Significant differences are indicated by absolute p values in the graph.

**Figure 7:**
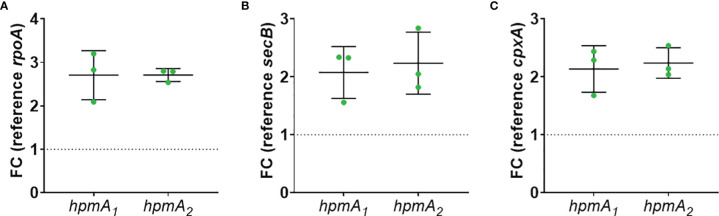
Increased expression of *P. mirabilis* hmpA upon stimulation with CCM. *C. albicans* was inoculated at 10^6^ CFUs/ml into cell culture medium and cultivated for 24 h at standard cell culture conditions. Resulting *Candida*-conditioned medium (CCM) and a medium control (KBM) were collected and sterile-filtered. *P. mirabilis* was inoculated at 10^5^ CFUs/ml into CCM or KBM and incubated for 5 h under standard cell culture conditions. Bacteria pellets were harvested and processed for qPCR analysis of hpmA gene expression using two primer sets: *hpmA*_1_ and *hpmA*_2_. Fold change of differential expression was calculated by Δ/ΔCt related to reference gene **(A)** *rpoA*, **(B)** *secB*, and **(C)** *cpxA*. Mean and SD of three independently performed experiments are plotted.

**Figure 8:**
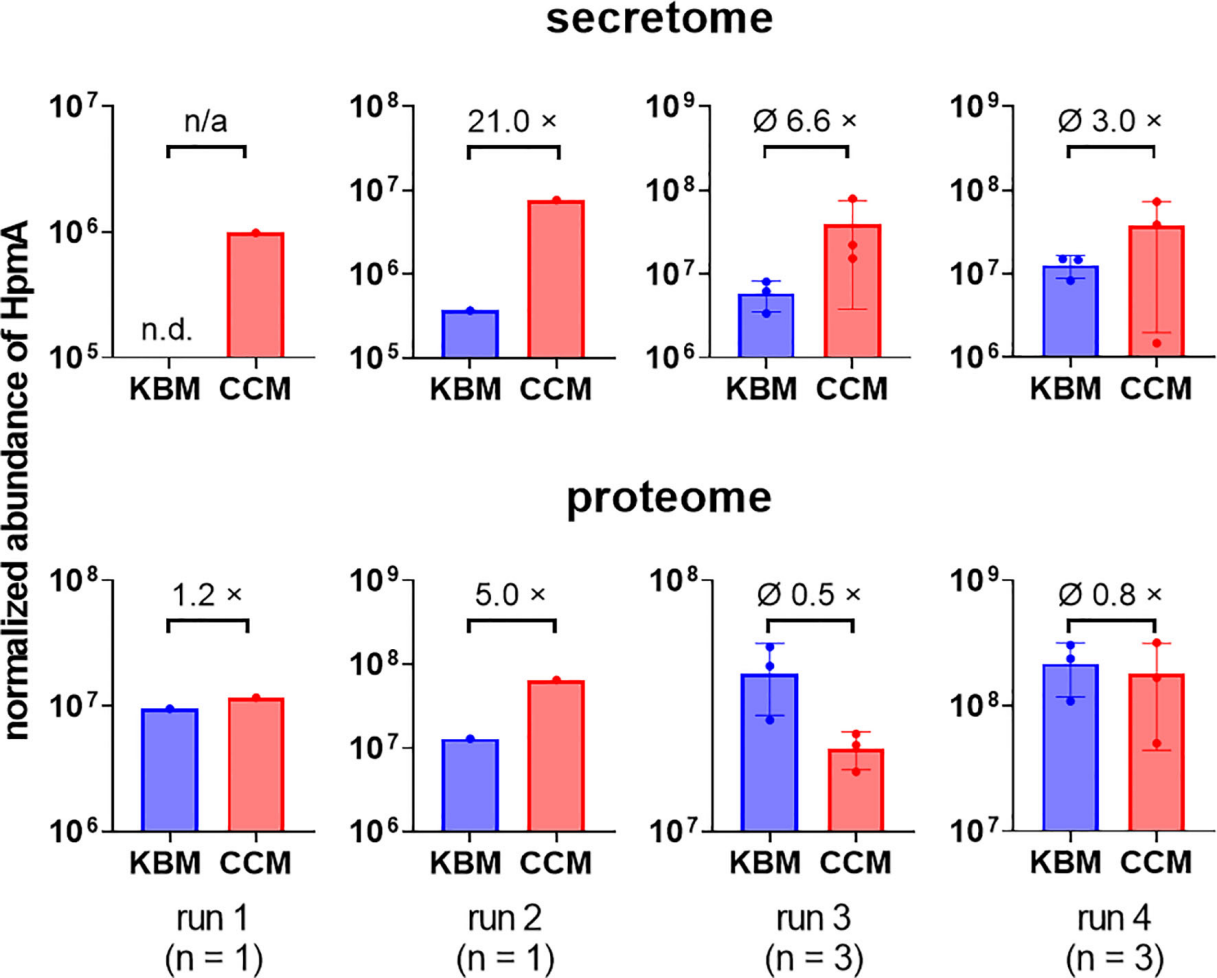
Increased relative abundance of *P. mirabilis* HpmA upon incubation in CCM. *C. albicans* was inoculated at 10^6^ CFUs/ml into cell culture medium and cultivated for 24 h at standard cell culture conditions. *Candida*-conditioned medium (CCM) and a medium control (KBM) was harvested and sterile-filtered. *P. mirabilis* was inoculated into CCM or regular cell culture medium KBM and incubated for 5 h under standard cell culture conditions. Bacteria pellets (proteome) and supernatants (secretome) were harvested and processed for subsequent LC-MS/MS analysis to quantify the relative abundance of HpmA. Run 1 and 2 were performed with one sample set at a time, run 3 and 4 were performed with 3 independently generated sample sets at a time. Relative abundances and, if applicable, means and SD are plotted (KBM: blue, CCM: red) as well as mean fold changes (×) of relative abundance, n.d. not detected, n/a not applicable. Using an unpaired Student’s t-test, relative HpmA abundance at conditions KBM and CCM were compared for run 3 and 4, no statistical significance was detected.

To test if soluble factors or alteration of medium composition by *C. albicans* are indeed contributing to the increased damage observed in transwell experiments, we prepared *Candida-*conditioned medium (CCM) by culturing the fungus in the cell culture medium KBM that was used for infection experiments. Sterile-filtered CCM was then used for *P. mirabilis* monoinfection. Compared to KBM, infection in CCM resulted in significantly higher cell damage (**Figure 6A**) that was not due to a direct cytotoxic effect of CCM (**Figure S3**) or differences in pH (Supp. Tab. 3). Since we established that the *P. mirabilis* hemolysin HmpA is essential for host cell damage during coinfection, we investigated if incubation in CCM led to increased transcription of *hmpA*. Moderately increased (approximately 2-fold) expression of *hmpA* was observed in CCM compared to KBM (**Figure 7**). Proteome and secretome analyses showed high variability of normalized HpmA abundance within and between experiments, but a trend towards higher production and secretion of HmpA in CCM compared to KBM was detectable in all experiments (**Figure 8**).

To gain further information on the molecules in CCM triggering the increased damage caused by *P. mirabilis*, size-exclusion filtration of CCM was performed. Here, the fraction below 3 kDa retained damage-inducing properties (**Figure 6A**). Furthermore, heat-treatment of CCM *prior* to the infection (72°C for 30 min) did not affect damage induction (**Figure S4A**), suggesting that small, heat-stable molecules such as metabolites might be responsible for the observed effect.

*C. albicans* is known to rapidly consume glucose (Chen et al., 2020), which is one of the main carbon sources in the cell culture medium used. We therefore quantified glucose in the culture media upon pre-incubation with *C. albicans* at the time point when *P. mirabilis* was typically added to the infection experiments (24 h). Medium of uninfected enterocyte cultures contained 4.5 ± 1.3 mM glucose (a 48% reduction from 8.5 ± 0.5 mM in fresh KBM), while no glucose was detectable in *C. albicans*-infected cultures and CCM. The lack of key nutrients is a known potential trigger for bacterial virulence (Rohmer et al., 2011). To test the role of glucose depletion during coinfection, we supplemented the medium with glucose (10 mM) immediately before addition of *P. mirabilis*. Although monoinfection damage by *C. albicans* increased with glucose supplementation, coinfection damage was significantly lower and the FDC reduced from 3.0 to 1.6 (**Figure 6B**). Similarly, *P. mirabilis*-induced host cell damage during monoinfection in CCM was reduced if glucose was added (p = 0.0622, **Figure 6C**). However, glucose supplementation of CCM and during coinfection did not fully reduce the damage to the respective controls (**Figures 6B, D**), suggesting that additional factors contribute to the effect of CCM.

The *P. mirabilis* urease converts urea to NH_3_ and CO_2_ and contributes to *Proteus* cytotoxicity. It is often expressed simultaneously with the hemolysin HpmA (Mobley et al., 1991; Allison et al., 1992b; Liaw et al., 2000; Wang et al., 2006). Incubation of *C. albicans* for 24 h led to a significant increase of urea in cell culture supernatants both in the absence (0.83 ± 0.05 mM *versus* 1.10 ± 0 mM) and presence of enterocytes (0.88 ± 0.10 mM *versus* 1.3 ± 0.10 mM). Based on this, we performed *P. mirabilis* monoinfections with increasing concentrations of urea, ranging from 0.5 mM to 30 mM. A statistical trend of dose-dependent damage increase was observed, but no significantly enhanced host cell damage was detected with even the highest concentration of 30 mM (p = 0.0549, **Figure 6D**). In addition, no major pH change, which would indicate urease-dependent production of NH_3_, was detected during enterocyte coinfections (Supp. Tab. 3). Thus, it is unlikely that the moderately increased amount of urea contributes significantly to increased damage in CCM.

The fungal quorum sensing molecule farnesol is produced by *C. albicans in vitro* at densities exceeding 10^6^ CFUs/ml, inhibits filamentation (Saidi et al., 2006; Polke et al., 2017) and has been shown to influence interactions of *C. albicans* with bacteria such as *Pseudomonas aeruginosa* and *Staphylococcus aureus* (Cugini et al., 2010; Vila et al., 2019). Hence, we performed germination assays using the *C. albicans eed1*Δ/Δ that is hypersensitive to the filamentation-inhibiting properties of farnesol as a proxy for farnesol detection (Polke et al., 2017). This experiment showed reduced filamentation of *C. albicans eed1*Δ/Δ in the presence of CCM but not KBM (**Figure S5**). In addition, we quantified farnesol in supernatants and cell pellets of *C. albicans* cultures prepared as described for CCM, and detected 0.262 ± 0.035 µM farnesol in supernatants. When *P. mirabilis* monoinfections were spiked with farnesol, a significant increase of host cell damage occurred only if 400 µM were added (**Figure 6E**). As this is clearly more than detected in *C. albicans* culture supernatant, we considered practical problems during farnesol spiking as a potential confounder. Due to its hydrophobic nature, farnesol tends to poorly dissolve in hydrophilic liquids and additionally adsorbs to plastic surfaces. Thus, the effective concentration achieved during the infection experiments might be smaller than the calculated concentration aimed for. To test this, we spiked KBM with 200 µM farnesol and quantified farnesol concentration immediately after spiking or after 24 h incubation under cell culture conditions. Only 4.9% (9.88 ± 2.28 µM) and 13.6% (27.15 ± 1.17 µM) of the farnesol could be detected, respectively. This suggests that there is a substantial difference between the farnesol concentration spiked into the infection and the actual effective concentration that *P. mirabilis* encounters. Hence, farnesol might be present in CCM at biologically potent concentrations and thereby affect *P. mirabilis* virulence. However, farnesol is clearly not the main factor in CCM promoting *P. mirabilis*-induced enterocyte damage.

### 3.6 The Impact of Coinfection Timing on Synergistic Damage

**Figure 9:**
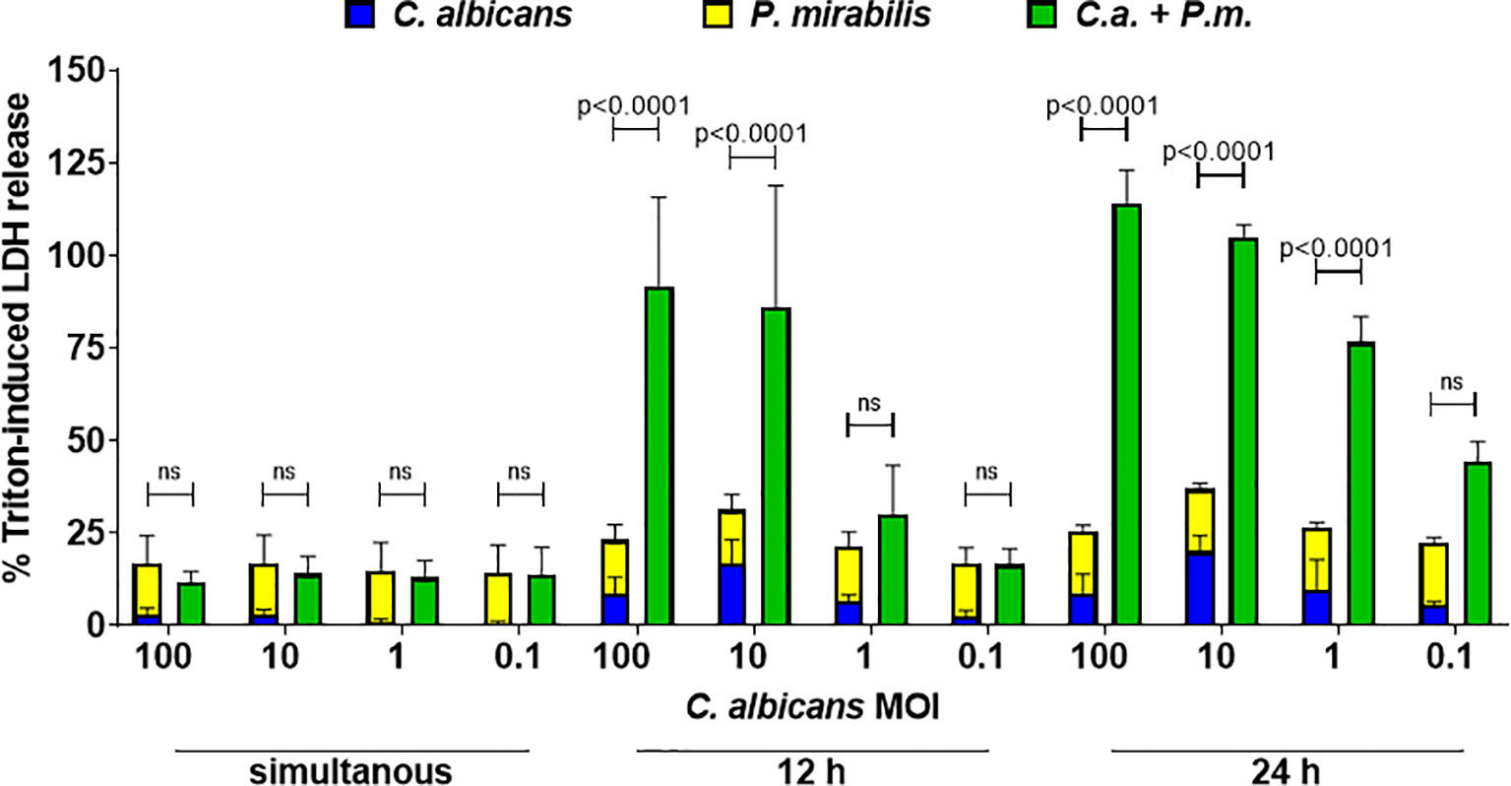
*Candida*-conditioned medium increases enterocyte damage caused by *P. mirabilis* infection. *C. albicans* SC5314 infection of enterocytes at MOI 100 to 0.1 was performed 24 h or 12 h before or at the same time as addition of *P. mirabilis* HI4320 at MOI 1. Host cell damage was assessed by LDH release 5 h after adding *P. mirabilis* related to a Triton-induced high control corrected for spontaneous cell death of uninfected enterocytes. Means and SD from at three independently performed experiments are plotted; the sum of monoinfection damage (*C. albicans*: blue, *P. mirabilis*: yellow; shown as stacked) was compared to coinfection damage (green) by 2-way ANOVA and Šídák’s multiple comparisons test. Coinfection damage with and without glucose was compared using unpaired two-sided t-test. Significant differences are indicated by absolute p values in the graph, ns, not significant.

In contrast to oral epithelial cells, LDH release during *in vitro* infection of enterocytes with *C. albicans* becomes only detectable after 12 h and increases towards 24 h after infection (Dalle et al., 2010). This can be explained by the requirement of adhesion, hyphae formation, and invasion as prerequisites for damage induction (Allert et al., 2018). The rapid proliferation of *P. mirabilis* (**Figure 1B**), in contrast, results in overgrowth of the cell culture over time. In addition, *P. mirabilis*-induced host cell damage increases rapidly, thereby limiting the time window for analysis (Mobley et al., 1991). Due to this, we decided to perform infection with *C. albicans* first, followed by addition of *P. mirabilis* after 24 h and analysis of damage after another 5 h. This does not necessarily reflect all situations *in vivo* in which both microbes are likely to be present also simultaneously. To address this point, we performed simultaneous infections, and infection with *P. mirabilis* 12 h after *C. albicans* infection. Synergistic damage was statistically evident if *P. mirabilis* was added 12 h after *C. albicans* at high *Candida* MOIs exceeding MOI 1, but not after simultaneous infection (**Figure 9**). As physical interactions between the microbes might require time to be established, and nutrient consumption/metabolite production is a time-dependent process, the 5 h observation period for simultaneous coinfection might be insufficient to establish the interactions and changes necessary for synergistic damage. We can, however, not exclude that the nature of interactions differs depending on the context, and that a pre-established infection of enterocytes by *C. albicans* is necessary to promote synergistic damage by *P. mirabilis*.

## 4 Discussion

The yeast *C. albicans* and the Gram-negative bacterium *P. mirabilis* share multiple mucosal niches in the human body, including the gut. Here we show that subsequent infection of enterocytes with the yeast *C. albicans* and the Gram-negative bacterium *P. mirabilis* leads to enhanced host cell damage mediated by *Proteus* hemolysin HpmA. *C. albicans* triggers *P. mirabilis* virulence by contact-dependent and contact-independent, soluble factors that are released or consumed during multifactorial niche modifications. Glucose consumption as well as the production of urea and farnesol might be among the clues that *P. mirabilis* reacts to during coinfection.

*P. mirabilis*-induced damage and the synergistic effect during coinfection was absent in two independently generated hemolysin-deficient *P. mirabilis* mutants (Mobley et al., 1991; Seo et al., 2015), indicating that the hemolysin HpmA is the key effector mediating enterocyte cytotoxicity. We furthermore observed moderately increased *hpmA* gene expression as well as increased relative abundance of secreted hemolysin during cultivation of *P. mirabilis* in CCM. Given that the use of CCM led to a significant increase of *P. mirabilis*-induced damage which, however, not reached the level of damage during coinfection, it appears possible that hemolysis production is further increased if contact with fungi occurs. Direct testing of this hypothesis turned out to be technically difficult as isolation of sufficient amounts of bacterial mRNA and direct hemolysin detection in actual coinfection samples were challenging. Another mechanism leading to increased coinfection damage could be increased contact of bacteria with host cells mediated by the physical interactions between bacteria and fungi: Generally, HpmA is described as an Ca-independent extracellular hemolysin that mediates hemolytic activity of cell-free *P. mirabilis* supernatants (Swihart and Welch, 1990a; Swihart and Welch, 1990b; Uphoff and Welch, 1990; Weaver et al., 2009). Its secretion and activation depend on the facilitator protein HpmB (Uphoff and Welch, 1990; Jacob-Dubuisson et al., 1997). However, hemolysis of *P. mirabilis* is typically measured directly with intact bacteria or by applying whole-cell preparations to erythrocytes (Koronakis et al., 1987; Mobley et al., 1991). This might imply that although the *P. mirabilis* hemolysin is secreted, it is most potent if released in close proximity to target cells, *e.g.* due to concentration effects. In this case, binding to fungi could increase the local concentration of *P. mirabilis* hemolysin on the enterocytes, resulting in increased cytotoxicity.

We did not aim to identify distinct factors that mediate binding of *P. mirabilis* to *C. albicans*. However, the observation that not only *C. albicans* but also other yeast species promote synergistically enhanced enterocyte damage could indicate conserved factors or unspecific binding. Interestingly, heat-inactivation of *C. albicans* yeast cells completely abolished the synergistic effect. Since non-filamentous *C. albicans* mutants induced synergistic damage, the lack of hyphal development cannot explain why inactivated yeast failed to induce synergism. The results with non-filamentous *C. albicans* mutants and other fungal species, such as *S. cerevisiae*, also excludes interaction *via* hypha-specific epitopes as a requirement for synergism. Of note, *C. parapsilosis* did not induce synergism. During enterocyte infection, *C. parapsilosis* did reduce glucose to undetectable levels, comparable to *C. albicans*, excluding reduced glucose consumption as an explanation for the observed difference. Other factors might be differences in farnesol production or other metabolites, or differences in the physical interaction between *Candida* and *P. mirabilis*. While not further investigated in this study, the differences observed between *C. parapsilosis* and the other fungal species might be useful in the future to identify additional fungal factors contributing to synergistic damage.

How expression of the *Proteus* hemolysin HpmA and the facilitator protein HpmB is regulated, is only incompletely understood, but it is linked to swarming. Swarming is typically observed on solid surfaces and it requires the differentiation of rod-shaped vegetative cells (also called swimmers) into elongated hyperflagellated swarm cells (Belas and Suvanasuthi, 2005; Szostek and Rather, 2013; Tuson et al., 2013; Little et al., 2019). It is associated with increased expression of virulence effectors, including hemolysin (Allison et al., 1992b; Gygi et al., 1995; Liaw et al., 2000; Fraser et al., 2002; Jones et al., 2004; Belas and Suvanasuthi, 2005; Wang et al., 2006). Swarming cells show augmented invasion into urothelial cells, independent of hemolysin, and the higher expression of hemolysin makes them more cytotoxic than vegetative cells (Allison et al., 1992a). A connection between swarming, hemolysin expression, and host cell damage is also implied by our results: The swarming inhibitor PNPG (Williams, 1973; Koronakis et al., 1987) reduced both *P. mirabilis* mono- and coinfection damage. This is consistent not only with a role of swarming for virulence, but also findings that PNPG inhibits virulence gene expression, including hemolysin (Liaw et al., 2000; Jones et al., 2009). Furthermore, deletion of *fliF*, encoding a component of the flagellar basal body (Himpsl et al., 2008), resulted in reduced damage in both, *P. mirabilis* mono- and coinfection. Similar to *fliF*, *fliL* encodes a flagellar basal body component (Belas and Suvanasuthi, 2005). Deletion of *fliL* results in development of non-motile hyperelongated swarmer cells and “inappropriate swarmer cells” in otherwise non-inducing liquid conditions, and upregulation of the hemolysin facilitator gene *hpmB* (Belas and Suvanasuthi, 2005). In our study, however, we observed only *P. mirabilis* rods, not elongated swarmer cells, binding to *Candida* in SEM imaging of coincubation and coinfection. Thus, whether swarming regulators are directly involved in the induction of synergistic damage during *C. albicans- P. mirabilis* coinfections remains unclear.

Although physical interactions were clearly required for full synergistic damage, conditioning of the medium by *C. albicans* also induced more damage by *P. mirabilis*. Similarly, metabolic cross-talk with other bacteria was shown to alter *P. mirabilis* virulence in the context of *in vivo* urinary infections (Armbruster et al., 2017a; Armbruster et al., 2017b). In these cases, urease acted as the main virulence factor, which prompted us to determine whether urea production by *Candida* might contribute to synergistic host cell damage in our model. While high doses of urea led to a significant increase of *P. mirabilis*-induced damage, the necessary concentration was higher than in CCM. We furthermore observed that glucose depletion and farnesol production by *C. albicans* induced increased *P. mirabilis*-mediated damage. Increased expression of virulence factors upon glucose deprivation has been described for different bacterial pathogens, including *E. coli*, *Salmonella* Typhi, *Klebsiella oxytoca*, and *Yersinia pestis* (Jofre et al., 2014; Haycocks et al., 2015; Ritzert and Lathem, 2018; Rodriguez-Valverde et al., 2021). Cross-kingdom signaling properties of farnesol have been described for coinfections of *C. albicans* and *Staphylocccus aureus* where farnesol enhanced *S. aureus* biofilm formation and reduced staphyloxanthin production (Kong et al., 2017). Given that neither urea, glucose nor farnesol alone fully recapitulated the effect observed with CCM, it appears likely that not a single factor but the combination of nutrient depletion and metabolite production by *C. albicans*, including factors not addressed in this study, drives increased virulence of *P. mirabilis* during coinfection. This also explains why synergistic coinfection damage was only observed if *C. albicans* was incubated with host cells for a prolonged time, and only with viable fungal cells, as media composition can be expected to progressively change over time by fungal metabolic activity. Finally, while we did not observe a direct negative effect of CCM on host cell viability, we cannot exclude that the altered nutrient/metabolite pattern in CCM compared to KBM negatively affects host cell resilience to the *P. mirabilis* hemolysin.

It should be noted that the prevalence of *P. mirabilis* in the gut is generally low, but might increase in various inflammatory diseases (Kanareykina et al., 1987; Elias-Oliveira et al., 2020; Zhang et al., 2021). If this leads to situations of significant overgrowth of both *P. mirabilis* and *C. albicans* in the same patient, remains unclear. Thus, the results obtained in this study might not be fully translatable to clinical situations; however, it expands our knowledge on the mechanisms behind and consequences of fungal-bacterial interactions within the host. A clinically relevant situation, however, is co-occurrence of *P. mirabilis* and *C. albicans* in the urinary tract. Although *C. albicans* is not considered a cause of UTI, the fungus is frequently isolated from urine ( (Kauffman, 2005; Kauffman et al., 2011; Ackerman and Underhill, 2017; Jacobs et al., 2018), and its presence might aggravate bacterial UTI caused by *P. mirabilis*.

In summary, our study provides evidence that *C. albicans* is able to provide a virulence-triggering local niche for *P. mirabilis* that augments enterocyte damage mediated by the *Proteus* hemolysin. While the exact signaling pathways are still unknown, our work emphasizes the potential of cross-kingdom interactions to locally alter microbial virulence and thereby the importance of comprehensive microbial diagnosis and assessment of patients harboring a potentially risky low-diversity microbiota upon medical intervention and antibiosis.

## Data Availability Statement

The datasets presented in this study can be found in online repositories. The names of the repository/repositories and accession number(s) can be found below: https://www.ebi.ac.uk/pride/archive/projects/PXD031222.

## Author Contributions

Authors have contributed as follows: Conceptualization, project administration and manuscript draft by MN and IJ. Investigation and methodology by MJN, MK, MH, KD, TK, TGK, IA, FK, SN and SL. Data curation by MJN, MH, KD, TK, TGK, IA, FK, SN, SL. Formal analysis by MJN, TK and TGK. Methodology and resources by CA, HM, TM, IDJ. Validation by IDJ. Supervision by MJN, OK, AB and IDJ. Funding acquisition by OK, AB, IDJ. All authors revised and approved the manuscript.

## Funding

The work was financially supported by the German Federal Ministry for Education and Research (BMBF) through the Center of Sepsis Control and Care, and the German Research Foundation (DFG) through the TRR 124 FungiNet, “Pathogenic fungi and their human host: Networks of Interaction,” DFG project number 210879364, projects C5 (to IJ) and Z2 (to OK). Further, the authors acknowledge support by the Jena School for Microbial Communication (JSMC).

## Conflict of Interest

The authors declare that the research was conducted in the absence of any commercial or financial relationships that could be construed as a potential conflict of interest.

## Publisher’s Note

All claims expressed in this article are solely those of the authors and do not necessarily represent those of their affiliated organizations, or those of the publisher, the editors and the reviewers. Any product that may be evaluated in this article, or claim that may be made by its manufacturer, is not guaranteed or endorsed by the publisher.

## References

[B1] Ackerman A. L. Underhill D. M. (2017). The Mycobiome of the Human Urinary Tract: Potential Roles for Fungi in Urology. Ann. Transl. Med. 5 (2), 31. doi: 10.21037/atm.2016.12.69 28217696 PMC5300854

[B2] Alamuri P. Eaton K. A. Himpsl S. D. Smith S. N. Mobley H. L. (2009). Vaccination With Proteus Toxic Agglutinin, A Hemolysin-Independent Cytotoxin *In Vivo*, Protects Against *Proteus Mirabilis* Urinary Tract Infection. Infect. Immun. 77 (2), 632–641. doi: 10.1128/IAI.01050-08 19029299 PMC2632039

[B3] Allert S. Forster T. M. Svensson C. M. Richardson J. P. Pawlik T. Hebecker B.. (2018). *Candida Albicans*-Induced Epithelial Damage Mediates Translocation Through Intestinal Barriers. mBio 9 (3), e00915–18. doi: 10.1128/mBio.00915-18 29871918 PMC5989070

[B4] Allison C. Coleman N. Jones P. L. Hughes C. (1992a). Ability of *Proteus Mirabilis* to Invade Human Urothelial Cells is Coupled to Motility and Swarming Differentiation. Infect. Immun. 60 (11), 4740–4746. doi: 10.1128/iai.60.11.4740-4746.1992 1398984 PMC258226

[B5] Allison C. Lai H. C. Hughes C. (1992b). Co-Ordinate Expression of Virulence Genes During Swarm-Cell Differentiation and Population Migration of *Proteus Mirabilis* . Mol. Microbiol. 6 (12), 1583–1591. doi: 10.1111/j.1365-2958.1992.tb00883.x 1495387

[B6] Alteri C. J. Himpsl S. D. Mobley H. L. (2015). Preferential Use of Central Metabolism *In Vivo* Reveals a Nutritional Basis for Polymicrobial Infection. PloS Pathog. 11 (1), e1004601. doi: 10.1371/journal.ppat.1004601 25568946 PMC4287612

[B7] Armbruster C. E. Forsyth-DeOrnellas V. Johnson A. O. Smith S. N. Zhao L. Wu W.. (2017a). Genome-Wide Transposon Mutagenesis of *Proteus Mirabilis*: Essential Genes, Fitness Factors for Catheter-Associated Urinary Tract Infection, and the Impact of Polymicrobial Infection on Fitness Requirements. PloS Pathog. 13 (6), e1006434. doi: 10.1371/journal.ppat.1006434 28614382 PMC5484520

[B8] Armbruster C. E. Mobley H. L. (2012). Merging Mythology and Morphology: The Multifaceted Lifestyle of *Proteus Mirabilis* . Nat. Rev. Microbiol. 10 (11), 743–754. doi: 10.1038/nrmicro2890 23042564 PMC3621030

[B9] Armbruster C. E. Mobley H. L. T. Pearson M. M. (2018). Pathogenesis of *Proteus Mirabilis* Infection. EcoSal. Plus. 8 (1), 10.1128/ecosalplus.ESP-0009-2017. doi: 10.1128/ecosalplus.ESP-0009-2017 PMC5880328 29424333

[B10] Armbruster C. E. Smith S. N. Johnson A. O. DeOrnellas V. Eaton K. A. Yep A.. (2017b). The Pathogenic Potential of *Proteus Mirabilis* Is Enhanced by Other Uropathogens During Polymicrobial Urinary Tract Infection. Infect. Immun. 85 (2), e00808–16. doi: 10.1128/IAI.00808-16 27895127 PMC5278182

[B11] Armstrong H. Bording-Jorgensen M. Dijk S. Wine E. (2018). The Complex Interplay Between Chronic Inflammation, the Microbiome, and Cancer: Understanding Disease Progression and What We Can do to Prevent it. Cancers (Basel). 10 (3), 83. doi: 10.3390/cancers10030083 PMC5876658 29558443

[B12] Belas R. Suvanasuthi R. (2005). The Ability of *Proteus Mirabilis* to Sense Surfaces and Regulate Virulence Gene Expression Involves FliL, A Flagellar Basal Body Protein. J. Bacteriol. 187 (19), 6789–6803. doi: 10.1128/JB.187.19.6789-6803.2005 16166542 PMC1251568

[B13] Brauer A. L. White A. N. Learman B. S. Johnson A. O. Armbruster C. E. (2019). D-Serine Degradation by *Proteus Mirabilis* Contributes to Fitness During Single-Species and Polymicrobial Catheter-Associated Urinary Tract Infection. mSphere 4 (1), e00020–19. doi: 10.1128/mSphere.00020-19 30814316 PMC6393727

[B14] Cestari S. E. Ludovico M. S. Martins F. H. da Rocha S. P. Elias W. P. Pelayo J. S. (2013). Molecular Detection of HpmA and HlyA Hemolysin of Uropathogenic *Proteus Mirabilis* . Curr. Microbiol. 67 (6), 703–707. doi: 10.1007/s00284-013-0423-5 23884594

[B15] Chen X. Zhang Z. Chen Z. Li Y. Su S. Sun S. (2020). Potential Antifungal Targets Based on Glucose Metabolism Pathways of *Candida Albicans* . Front. Microbiol. 11. doi: 10.3389/fmicb.2020.00296 PMC7093590 32256459

[B16] Cugini C. Morales D. K. Hogan D. A. (2010). *Candida Albicans*-Produced Farnesol Stimulates Pseudomonas Quinolone Signal Production in LasR-defective Pseudomonas Aeruginosa Strains. Microbiol. (Reading). 156 (Pt 10), 3096–3107. doi: 10.1099/mic.0.037911-0 PMC3068698 20656785

[B17] Dalle F. Wachtler B. L’Ollivier C. Holland G. Bannert N. Wilson D.. (2010). Cellular Interactions of *Candida Albicans* With Human Oral Epithelial Cells and Enterocytes. Cell Microbiol. 12 (2), 248–271. doi: 10.1111/j.1462-5822.2009.01394.x 19863559

[B18] Dekaboruah E. Suryavanshi M. V. Chettri D. Verma A. K. (2020). Human Microbiome: An Academic Update on Human Body Site Specific Surveillance and its Possible Role. Arch. Microbiol. 202 (8), 2147–2167. doi: 10.1007/s00203-020-01931-x 32524177 PMC7284171

[B19] Eggimann P. Que Y. A. Revelly J. P. Pagani J. L. (2015). Preventing Invasive Candida Infections. Where Could We Do Better? J. Hosp. Infect. 89 (4), 302–308. doi: 10.1016/j.jhin.2014.11.006 25592726

[B20] Elias-Oliveira J. Leite J. A. Pereira I. S. Guimaraes J. B. Manso G. Silva J. S.. (2020). NLR and Intestinal Dysbiosis-Associated Inflammatory Illness: Drivers or Dampers? Front. Immunol. 11. doi: 10.3389/fimmu.2020.01810 PMC7438795 32903730

[B21] Fan D. Coughlin L. A. Neubauer M. M. Kim J. Kim M. S. Zhan X.. (2015). Activation of HIF-1alpha and LL-37 by Commensal Bacteria Inhibits *Candida Albicans* Colonization. Nat. Med. 21 (7), 808–814. doi: 10.1038/nm.3871 26053625 PMC4496259

[B22] Fraser G. M. Claret L. Furness R. Gupta S. Hughes C. (2002). Swarming-Coupled Expression of the *Proteus Mirabilis hpmBA* Haemolysin Operon. Microbiol. (Reading). 148 (Pt 7), 2191–2201. doi: 10.1099/00221287-148-7-2191 PMC2528290 12101306

[B23] Gong D. Gong X. Wang L. Yu X. Dong Q. (2016). Involvement of Reduced Microbial Diversity in Inflammatory Bowel Disease. Gastroenterol. Res. Pract. 2016, 6951091. doi: 10.1155/2016/6951091 28074093 PMC5198157

[B24] Gouba N. Drancourt M. (2015). Digestive Tract Mycobiota: A Source of Infection. Med. Mal. Infect. 45 (1-2), 9–16. doi: 10.1016/j.medmal.2015.01.007 25684583

[B25] Gow N. A. van de Veerdonk F. L. Brown A. J. Netea M. G. (2012). C*andida Albicans* Morphogenesis and Host Defence: Discriminating Invasion From Colonization. Nat. Rev. Microbiol. 10 (2), 112–122. doi: 10.1038/nrmicro2711 PMC3624162 22158429

[B26] Gygi D. Bailey M. J. Allison C. Hughes C. (1995). Requirement for FlhA in Flagella Assembly and Swarm-Cell Differentiation by *Proteus Mirabilis* . Mol. Microbiol. 15 (4), 761–769. doi: 10.1111/j.1365-2958.1995.tb02383.x 7783646

[B27] Hamilton A. L. Kamm M. A. Ng S. C. Morrison M. (2018). *Proteus* Spp. As Putative Gastrointestinal Pathogens. Clin. Microbiol. Rev. 31 (3), e00085–17. doi: 10.1128/CMR.00085-17 29899011 PMC6056842

[B28] Haycocks J. R. Sharma P. Stringer A. M. Wade J. T. Grainger D. C. (2015). The Molecular Basis for Control of ETEC Enterotoxin Expression in Response to Environment and Host. PloS Pathog. 11 (1), e1004605. doi: 10.1371/journal.ppat.1004605 25569153 PMC4287617

[B29] Himpsl S. D. Lockatell C. V. Hebel J. R. Johnson D. E. Mobley H. L. T. (2008). Identification of Virulence Determinants in Uropathogenic *Proteus Mirabilis* Using Signature-Tagged Mutagenesis. J. Med. Microbiol. 57 (Pt 9), 1068–1078. doi: 10.1099/jmm.0.2008/002071-0 18719175

[B30] Hosseinzadeh A. Urban C. F. (2013). Novel Insight Into Neutrophil Immune Responses by Dry Mass Determination of *Candida Albicans* Morphotypes. PloS One 8 (10), e77993. doi: 10.1371/journal.pone.0077993 24205058 PMC3813559

[B31] Iacob S. Iacob D. G. (2019). Infectious Threats, the Intestinal Barrier, and Its Trojan Horse: Dysbiosis. Front. Microbiol. 10, 1676. doi: 10.3389/fmicb.2019.01676 31447793 PMC6692454

[B32] Jacob-Dubuisson F. Buisine C. Willery E. Renauld-Mongenie G. Locht C. (1997). Lack of Functional Complementation Between *Bordetella Pertussis* Filamentous Hemagglutinin and Proteus Mirabilis HpmA Hemolysin Secretion Machineries. J. Bacteriol. 179 (3), 775–783. doi: 10.1128/jb.179.3.775-783.1997 9006033 PMC178760

[B33] Jacobs D. M. Dilworth T. J. Beyda N. D. Casapao A. M. Bowers D. R. (2018). Overtreatment of Asymptomatic Candiduria Among Hospitalized Patients: A Multi-institutional Study. Antimicrob. Agents Chemother. 62 (1), e01464–17. doi: 10.1128/AAC.01464-17 29109159 PMC5740329

[B34] Jacobsen S. M. Shirtliff M. E. (2011). *Proteus Mirabilis* Biofilms and Catheter-Associated Urinary Tract Infections. Virulence 2 (5), 460–465. doi: 10.4161/viru.2.5.17783 21921687

[B35] Jofre M. R. Rodriguez L. M. Villagra N. A. Hidalgo A. A. Mora G. C. Fuentes J. A. (2014). Rpos Integrates CRP, Fis, and PhoP Signaling Pathways to Control *Salmonella Typhi* Hlye Expression. BMC Microbiol. 14, 139. doi: 10.1186/1471-2180-14-139 24885225 PMC4105832

[B36] Jones S. M. Dang T. T. Martinuzzi R. (2009). Use of Quorum Sensing Antagonists to Deter the Formation of Crystalline *Proteus Mirabilis* Biofilms. Int. J. Antimicrob. Agents 34 (4), 360–364. doi: 10.1016/j.ijantimicag.2009.06.011 19619987

[B37] Jones B. V. Young R. Mahenthiralingam E. Stickler D. J. (2004). Ultrastructure of *Proteus Mirabilis* Swarmer Cell Rafts and Role of Swarming in Catheter-Associated Urinary Tract Infection. Infect. Immun. 72 (7), 3941–3950. doi: 10.1128/IAI.72.7.3941-3950.2004 15213138 PMC427392

[B38] Juarez G. E. Mateyca C. Galvan E. M. (2020). *Proteus Mirabilis O*utcompetes *Klebsiella Pneumoniae* in Artificial Urine Medium Through Secretion of Ammonia and Other Volatile Compounds. Heliyon 6 (2), e03361. doi: 10.1016/j.heliyon.2020.e03361 32055744 PMC7005574

[B39] Kanareykina S. K. Misautova A. A. Zlatkina A. R. Levina E. N. (1987). *Proteus* Dysbioses in Patients With Ulcerative Colitis. Nahrung 31 (5-6), 557–561. doi: 10.1002/food.19870310570 3657933

[B40] Kapitan M. Niemiec M. J. Steimle A. Frick J. S. Jacobsen I. D. (2019). Fungi as Part of the Microbiota and Interactions With Intestinal Bacteria. Curr. Top. Microbiol. Immunol. 422, 265–301. doi: 10.1007/82_2018_117 30062595

[B41] Kauffman C. A. (2005). Candiduria. Clin. Infect. Dis. 41 Suppl 6, S371–S376. doi: 10.1086/430918 16108001

[B42] Kauffman C. A. Fisher J. F. Sobel J. D. Newman C. A. (2011). *Candida* Urinary Tract Infections–Diagnosis. Clin. Infect. Dis. 52 Suppl 6, S452–S456. doi: 10.1093/cid/cir111 21498838

[B43] Koehler P. Stecher M. Cornely O. A. Koehler D. Vehreschild M. Bohlius J.. (2019). Morbidity and Mortality of Candidaemia in Europe: An Epidemiologic Meta-Analysis. Clin. Microbiol. Infect. 25 (10), 1200–1212. doi: 10.1016/j.cmi.2019.04.024 31039444

[B44] Kong E. F. Tsui C. Kucharikova S. Van Dijck P. Jabra-Rizk M. A. (2017). Modulation of *Staphylococcus Aureus* Response to Antimicrobials by the *Candida Albicans* Quorum Sensing Molecule Farnesol. Antimicrob. Agents Chemother. 61 (12), e01573–17. doi: 10.1128/AAC.01573-17 28893777 PMC5700358

[B45] Koronakis V. Cross M. Senior B. Koronakis E. Hughes C. (1987). The Secreted Hemolysins of P*roteus Mirabilis*, *Proteus Vulgaris*, and *Morganella Morganii* are Genetically Related to Each Other and to the Alpha-Hemolysin of Escherichia Coli. J. Bacteriol. 169 (4), 1509–1515. doi: 10.1128/jb.169.4.1509-1515.1987 3549692 PMC211976

[B46] Kruger W. Vielreicher S. Kapitan M. Jacobsen I. D. Niemiec M. J. (2019). Fungal-Bacterial Interactions in Health and Disease. Pathogens 8 (2), 70. doi: 10.3390/pathogens8020070 PMC6630686 31117285

[B47] Lagunes L. Rello J. (2016). Invasive Candidiasis: From Mycobiome to Infection, Therapy, and Prevention. Eur. J. Clin. Microbiol. Infect. Dis. 35(8), 1221–6. doi: 10.1007/s10096-016-2658-0 27146877

[B48] Liaw S. J. Lai H. C. Ho S. W. Luh K. T. Wang W. B. (2000). Inhibition of Virulence Factor Expression and Swarming Differentiation in *Proteus Mirabilis* by P-Nitrophenylglycerol. J. Med. Microbiol. 49 (8), 725–731. doi: 10.1099/0022-1317-49-8-725 10933258

[B49] Little K. Austerman J. Zheng J. Gibbs K. A. (2019). Cell Shape and Population Migration Are Distinct Steps of *Proteus Mirabilis* Swarming That Are Decoupled on High-Percentage Agar. J. Bacteriol. 201 (11), e00726–18. doi: 10.1128/JB.00726-18 30858303 PMC6509654

[B50] Livak K. J. Schmittgen T. D. (2001). Analysis of Relative Gene Expression Data Using Real-Time Quantitative PCR and the 2(-Delta Delta C(T)) Method. Methods 25 (4), 402–408. doi: 10.1006/meth.2001.1262 11846609

[B51] Miranda L. N. van der Heijden I. M. Costa S. F. Sousa A. P. Sienra R. A. Gobara S.. (2009). *Candida* Colonisation as a Source for Candidaemia. J. Hosp. Infect. 72 (1), 9–16. doi: 10.1016/j.jhin.2009.02.009 19303662

[B52] Mirhakkak M. H. Schauble S. Klassert T. E. Brunke S. Brandt P. Loos D.. (2021). Metabolic Modeling Predicts Specific Gut Bacteria as Key Determinants for *Candida Albicans* Colonization Levels. ISME. J. 15 (5), 1257–1270. doi: 10.1038/s41396-020-00848-z 33323978 PMC8115155

[B53] Mobley H. L. Chippendale G. R. Swihart K. G. Welch R. A. (1991). Cytotoxicity of the HpmA Hemolysin and Urease of *Proteus Mirabilis* and *Proteus Vulgaris* Against Cultured Human Renal Proximal Tubular Epithelial Cells. Infect. Immun. 59 (6), 2036–2042. doi: 10.1128/IAI.59.6.2036-2042.1991 2037363 PMC257962

[B54] Mora Carpio A. L. Climaco A. (2021). “Fungemia Candidiasis,” (Treasure Island (FL: StatPearls).

[B55] Muller H. E. (1986). Occurrence and Pathogenic Role of *Morganella-Proteus-Providencia* Group Bacteria in Human Feces. J. Clin. Microbiol. 23 (2), 404–405. doi: 10.1128/JCM.23.2.404-405.1986 3517057 PMC268658

[B56] Niemiec M. J. Kapitan M. Polke M. Jacobsen I. D. (2017). “Commensal to Pathogen Transition of Candida Albicans,” (Esevier, Amsterdam, Netherlands: Reference Module in Life Sciences).

[B57] Pearson M. M. Sebaihia M. Churcher C. Quail M. A. Seshasayee A. S. Luscombe N. M.. (2008). Complete Genome Sequence of Uropathogenic *Proteus Mirabilis*, A Master of Both Adherence and Motility. J. Bacteriol. 190 (11), 4027–4037. doi: 10.1128/JB.01981-07 18375554 PMC2395036

[B58] Pearson M. M. Yep A. Smith S. N. Mobley H. L. (2011). Transcriptome of *Proteus Mirabilis* in the Murine Urinary Tract: Virulence and Nitrogen Assimilation Gene Expression. Infect. Immun. 79 (7), 2619–2631. doi: 10.1128/IAI.05152-11 21505083 PMC3191972

[B59] Peerbooms P. G. Verweij A. M. MacLaren D. M. (1984). Vero Cell Invasiveness of *Proteus Mirabilis* . Infect. Immun. 43 (3), 1068–1071. doi: 10.1128/iai.43.3.1068-1071.1984 6365782 PMC264295

[B60] Peerbooms P. G. Verweij A. M. MacLaren D. M. (1985). Uropathogenic Properties of *Proteus Mirabilis* and *Proteus Vulgaris* . J. Med. Microbiol. 19 (1), 55–60. doi: 10.1099/00222615-19-1-55 3881591

[B61] Peleg A. Y. Hogan D. A. Mylonakis E. (2010). Medically Important Bacterial-Fungal Interactions. Nat. Rev. Microbiol. 8 (5), 340–349. doi: 10.1038/nrmicro2313 20348933

[B62] Perez-Riverol Y. Csordas A. Bai J. Bernal-Llinares M. Hewapathirana S. Kundu D. J.. (2019). The PRIDE Database and Related Tools and Resources in 2019: Improving Support for Quantification Data. Nucleic Acids Res. 47 (D1), D442–D450. doi: 10.1093/nar/gky1106 30395289 PMC6323896

[B63] Polke M. Jacobsen I. D. (2017). Quorum Sensing by Farnesol Revisited. Curr. Genet. 63 (5), 791–797. doi: 10.1007/s00294-017-0683-x 28247023

[B64] Polke M. Leonhardt I. Kurzai O. Jacobsen I. D. (2018). Farnesol Signalling in *Candida Albicans* - More Than Just Communication. Crit. Rev. Microbiol. 44 (2), 230–243. doi: 10.1080/1040841X.2017.1337711 28609183

[B65] Polke M. Sprenger M. Scherlach K. Alban-Proano M. C. Martin R. Hertweck C.. (2017). A Functional Link Between Hyphal Maintenance and Quorum Sensing in *Candida Albicans* . Mol. Microbiol. 103 (4), 595–617. doi: 10.1111/mmi.13526 27623739

[B66] Ribet D. Cossart P. (2015). How Bacterial Pathogens Colonize Their Hosts and Invade Deeper Tissues. Microbes Infect. 17 (3), 173–183. doi: 10.1016/j.micinf.2015.01.004 25637951

[B67] Ritzert J. T. Lathem W. W. (2018). Depletion of Glucose Activates Catabolite Repression During Pneumonic Plague. J. Bacteriol. 200 (11), e00737–17. doi: 10.1128/JB.00737-17 29555700 PMC5952388

[B68] Rodriguez-Valverde D. Leon-Montes N. Soria-Bustos J. Martinez-Cruz J. Gonzalez-Ugalde R. Rivera-Gutierrez S.. (2021). Camp Receptor Protein Positively Regulates the Expression of Genes Involved in the Biosynthesis of *Klebsiella Oxytoca* Tilivalline Cytotoxin. Front. Microbiol. 12. doi: 10.3389/fmicb.2021.743594 PMC8515920 34659176

[B69] Rohmer L. Hocquet D. Miller S. I. (2011). Are Pathogenic Bacteria Just Looking for Food? Metabolism and Microbial Pathogenesis. Trends Microbiol. 19 (7), 341–348. doi: 10.1016/j.tim.2011.04.003 21600774 PMC3130110

[B70] Saidi S. Luitaud C. Rouabhia M. (2006). *In Vitro* Synergistic Effect of Farnesol and Human Gingival Cells Against Candida Albicans. Yeast 23 (9), 673–687. doi: 10.1002/yea.1389 16845684

[B71] Sanches M. S. Baptista A. A. S. de Souza M. Menck-Costa M. F. Koga V. L. Kobayashi R. K. T.. (2019). Genotypic and Phenotypic Profiles of Virulence Factors and Antimicrobial Resistance of *Proteus Mirabilis* Isolated From Chicken Carcasses: Potential Zoonotic Risk. Braz. J. Microbiol. 50 (3), 685–694. doi: 10.1007/s42770-019-00086-2 31049879 PMC6863274

[B72] Santus W. Devlin J. R. Behnsen J. (2021). Crossing Kingdoms: How the Mycobiota and Fungal-Bacterial Interactions Impact Host Health and Disease. Infect. Immun. 89 (4), e00648–20. doi: 10.1128/IAI.00648-20 33526565 PMC8090948

[B73] Seo S. U. Kamada N. Munoz-Planillo R. Kim Y. G. Kim D. Koizumi Y.. (2015). Distinct Commensals Induce Interleukin-1beta *Via* NLRP3 Inflammasome in Inflammatory Monocytes to Promote Intestinal Inflammation in Response to Injury. Immunity 42 (4), 744–755. doi: 10.1016/j.immuni.2015.03.004 25862092 PMC4408263

[B74] Shukla A. Sobel J. D. (2019). Vulvovaginitis Caused by *Candida* Species Following Antibiotic Exposure. Curr. Infect. Dis. Rep. 21 (11), 44. doi: 10.1007/s11908-019-0700-y 31707496

[B75] Sudbery P. E. (2011). Growth of *Candida Albicans* Hyphae. Nat. Rev. Microbiol. 9 (10), 737–748. doi: 10.1038/nrmicro2636 21844880

[B76] Swihart K. G. Welch R. A. (1990a). Cytotoxic Activity of the *Proteus Hemolysin* Hpma. Infect. Immun. 58 (6), 1861–1869. doi: 10.1128/iai.58.6.1861-1869.1990 2341182 PMC258736

[B77] Swihart K. G. Welch R. A. (1990b). The HpmA Hemolysin is More Common Than HlyA Among *Proteus* Isolates. Infect. Immun. 58 (6), 1853–1860. doi: 10.1128/iai.58.6.1853-1860.1990 2341181 PMC258735

[B78] Szostek B. A. Rather P. N. (2013). Regulation of the Swarming Inhibitor Disa in *Proteus Mirabilis* . J. Bacteriol. 195 (14), 3237–3243. doi: 10.1128/JB.00039-13 23687266 PMC3697635

[B79] Turner S. A. Butler G. (2014). The *Candida* Pathogenic Species Complex. Cold Spring Harb. Perspect. Med. 4 (9), a019778. doi: 10.1101/cshperspect.a019778 25183855 PMC4143104

[B80] Tuson H. H. Copeland M. F. Carey S. Sacotte R. Weibel D. B. (2013). Flagellum Density Regulates *Proteus Mirabilis* Swarmer Cell Motility in Viscous Environments. J. Bacteriol. 195 (2), 368–377. doi: 10.1128/JB.01537-12 23144253 PMC3553826

[B81] Uphoff T. S. Welch R. A. (1990). Nucleotide Sequencing of the P*roteus Mirabilis* Calcium-Independent Hemolysin Genes (Hpma and hpmB) Reveals Sequence Similarity With the *Serratia Marcescens* Hemolysin Genes (Shla and Shlb). J. Bacteriol. 172 (3), 1206–1216. doi: 10.1128/jb.172.3.1206-1216.1990 2407716 PMC208585

[B82] Vila T. Kong E. F. Ibrahim A. Piepenbrink K. Shetty A. C. McCracken C.. (2019). *Candida Albicans* Quorum-Sensing Molecule Farnesol Modulates Staphyloxanthin Production and Activates the Thiol-Based Oxidative-Stress Response in Staphylococcus Aureus. Virulence 10 (1), 625–642. doi: 10.1080/21505594.2019.1635418 31280653 PMC6629188

[B83] Wang W. B. Lai H. C. Hsueh P. R. Chiou R. Y. Lin S. B. Liaw S. J. (2006). Inhibition of Swarming and Virulence Factor Expression in *Proteus Mirabilis* by Resveratrol. J. Med. Microbiol. 55 (Pt 10), 1313–1321. doi: 10.1099/jmm.0.46661-0 17005777

[B84] Wang Q. Torzewska A. Ruan X. Wang X. Rozalski A. Shao Z.. (2010). Molecular and Genetic Analyses of the Putative *Proteus* O Antigen Gene Locus. Appl. Environ. Microbiol. 76 (16), 5471–5478. doi: 10.1128/AEM.02946-09 20581173 PMC2918944

[B85] Warren J. W. Tenney J. H. Hoopes J. M. Muncie H. L. Anthony W. C. (1982). A Prospective Microbiologic Study of Bacteriuria in Patients With Chronic Indwelling Urethral Catheters. J. Infect. Dis. 146 (6), 719–723. doi: 10.1093/infdis/146.6.719 6815281

[B86] Weaver T. M. Hocking J. M. Bailey L. J. Wawrzyn G. T. Howard D. R. Sikkink L. A.. (2009). Structural and Functional Studies of Truncated Hemolysin A From P*roteus Mirabilis* . J. Biol. Chem. 284 (33), 22297–22309. doi: 10.1074/jbc.M109.014431 19494116 PMC2755953

[B87] Williams F. D. (1973). Abolition of Swarming of *Proteus* by P-Nitrophenyl Glycerin: Application to Blood Agar Media. Appl. Microbiol. 25 (5), 751–754. doi: 10.1128/am.25.5.751-754.1973 4715553 PMC380906

[B88] Yeung Y. G. Stanley E. R. (2010). Rapid Detergent Removal From Peptide Samples With Ethyl Acetate for Mass Spectrometry Analysis. Curr. Protoc. Protein Sci. Chapter 16, Unit 16 12. doi: 10.1002/0471140864.ps1612s59 PMC2852680 20155730

[B89] Zaborin A. Smith D. Garfield K. Quensen J. Shakhsheer B. Kade M.. (2014). Membership and Behavior of Ultra-Low-Diversity Pathogen Communities Present in the Gut of Humans During Prolonged Critical Illness. MBio 5 (5), e01361–e01314. doi: 10.1128/mBio.01361-14 25249279 PMC4173762

[B90] Zhai B. Ola M. Rolling T. Tosini N. L. Joshowitz S. Littmann E. R.. (2020). High-Resolution Mycobiota Analysis Reveals Dynamic Intestinal Translocation Preceding Invasive Candidiasis. Nat. Med. 26 (1), 59–64. doi: 10.1038/s41591-019-0709-7 31907459 PMC7005909

[B91] Zhang J. Hoedt E. C. Liu Q. Berendsen E. Teh J. J. Hamilton A.. (2021). Elucidation of *Proteus Mirabilis* as a Key Bacterium in Crohn’s Disease Inflammation. Gastroenterology 160 (1), 317–330.e311. doi: 10.1053/j.gastro.2020.09.036 33011176

[B92] Zheng D. Liwinski T. Elinav E. (2020). Interaction Between Microbiota and Immunity in Health and Disease. Cell Res. 30 (6), 492–506. doi: 10.1038/s41422-020-0332-7 32433595 PMC7264227

