# Supplementary material for: Augmented Enterocyte Damage During Candida albicans and Proteus mirabilis Coinfection
Source: Front Cell Infect Microbiol. 2022 May 16;12:866416. doi: 10.3389/fcimb.2022.866416 (PMC9149288; doi:10.3389/fcimb.2022.866416)
Supplement: Supplementary file 1 [file DataSheet_1.pdf]

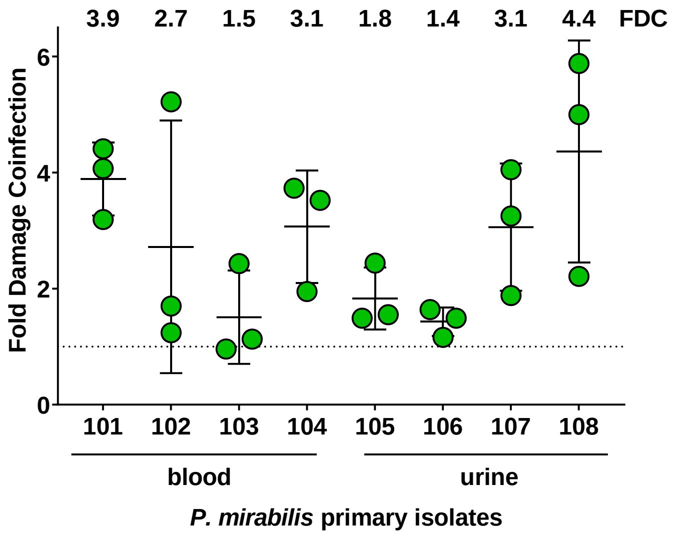

Figure S1: Different *P. mirabilis* clinical isolates lead to synergistic damage during coinfection.
Enterocytes were infected for 24 h with *C. albicans* SC5314 at MOI 10 and subsequently with different *P. mirabilis* primary clinical isolates derived from blood and urine at MOI 1 for 5 h. Host cell damage was assessed by quantification of LDH release and is shown as fold damage coinfection (FDC; relative damage induced by coinfection divided by the sum of damage induced by respective monoinfections) as a measure of synergistic potency. Values and SD from 3 independent experiments are plotted, mean fold damage coinfections (FDC) are indicated for each *P. mirabilis* isolate. Analysis by One-Way ANOVA with Tukey's multiple comparison test showed no statistically significant difference between the strains.

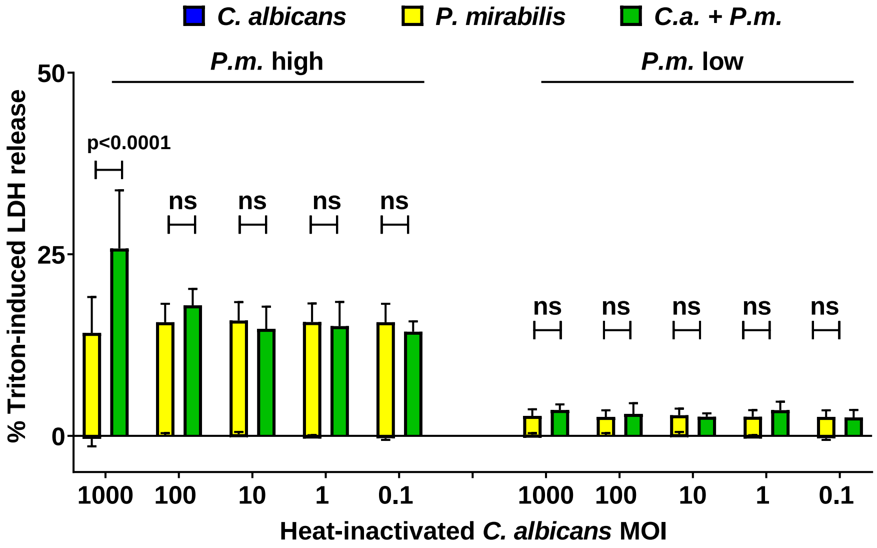

Figure S2: Heat-inactivation of *C. albicans* abolishes synergistic damage.
Enterocytes were infected for 24 h with heat-inactivated *C. albicans* SC5314 at MOI 1000 to 0.1 and subsequently with live *P. mirabilis* HI4320 at MOI 1 or 0.1 for 5 h. Host cell damage was assessed by LDH release related to a Triton-induced high control corrected for spontaneous cell death of uninfected enterocytes. Means and SD from 3 independent experiments are plotted (*C. albicans*: blue, *P. mirabilis*: yellow; shown as stacked); the sum of monoinfection damage was compared to coinfection damage (green) compared by Two-Way ANOVA and Šídák's multiple comparisons test. Significant differences are indicated by absolute p values in the graph, ns: not significant.

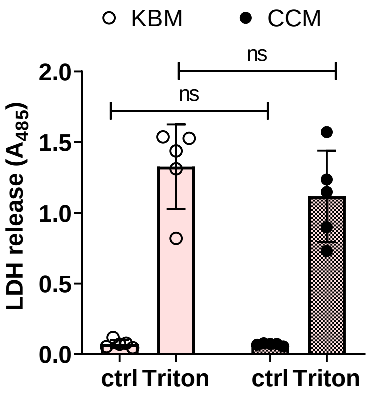

Figure S3: ***Candida*****-conditioned medium alone not cytotoxic**
Enterocyte toxicity of CCM in comparison to cell culture medium KBM was quantified by LDH release. As high damage reference, cells were lyzed with 0.25 % Triton. Means and SD of 5 independent experiments are plotted; CCM and KBM were compared by unpaired t-test; ns: not significant.

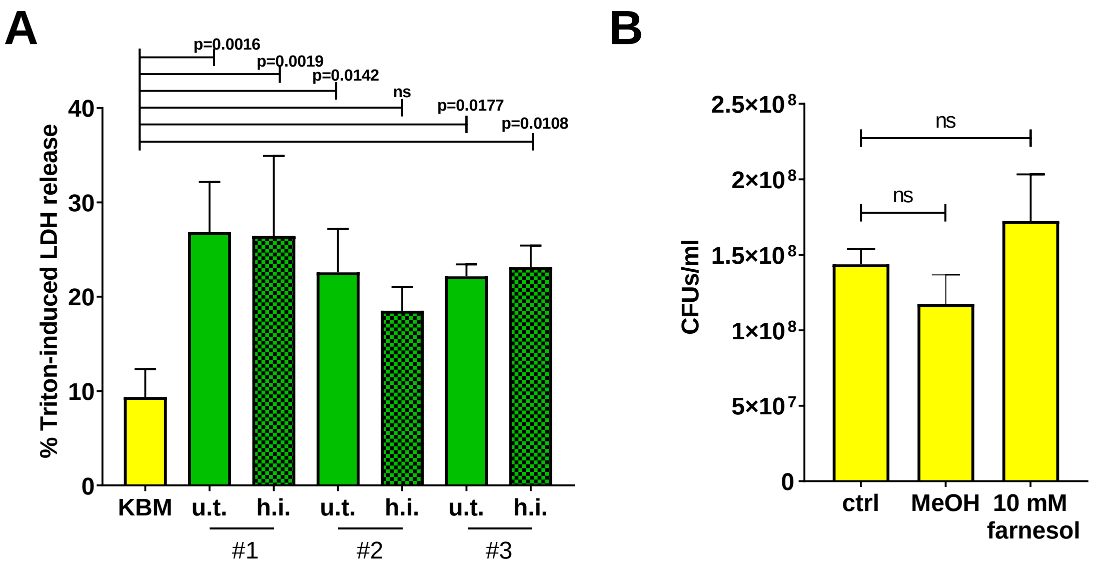

Figure S4: Heat-inactivation does not affect *Candida*-conditioned medium efficicacy and 10 mM farnesol does not reduce *P. mirabilis* viability

(A) *C. albicans* was inoculated at 10^6 CFUs/ml into cell culture medium and cultivated for 24 h at standard cell culture conditions. *Candida*-conditioned medium (CCM) and a medium control (KBM) were harvested, sterile-filtered, and (A) treated by heat-inactivation for 30 min at 72 °C. Heat-inactivated (h.i.) and corresponding untreated (u.t.) CCM produced on three independent occasions was compared to KBM. Means and SD from three independent experiments using independent preparations of CCM are depicted. Host cell damage in all conditions was compared to the enterocytes incubated with KBM for 24 h by One-Way ANOVA with Dunnett's multiple comparison test; * $p < 0.05$, ** $p < 0.01$, *** $p < 0.001$, comparisons yielding not significant are not indicated. **(B)** *Proteus mirabilis* was grown in KBM with added 10 mM farnesol, methanol, or without additives for 5 h and bacterial viability was determined by plating on CLED agar. *Proteus mirabilis* CFUs/ml of three independent experiments are plotted as mean and SD. Using the same statistical method as for (A), no statistical significance (ns) could be detected between the untreated control and the other conditions.

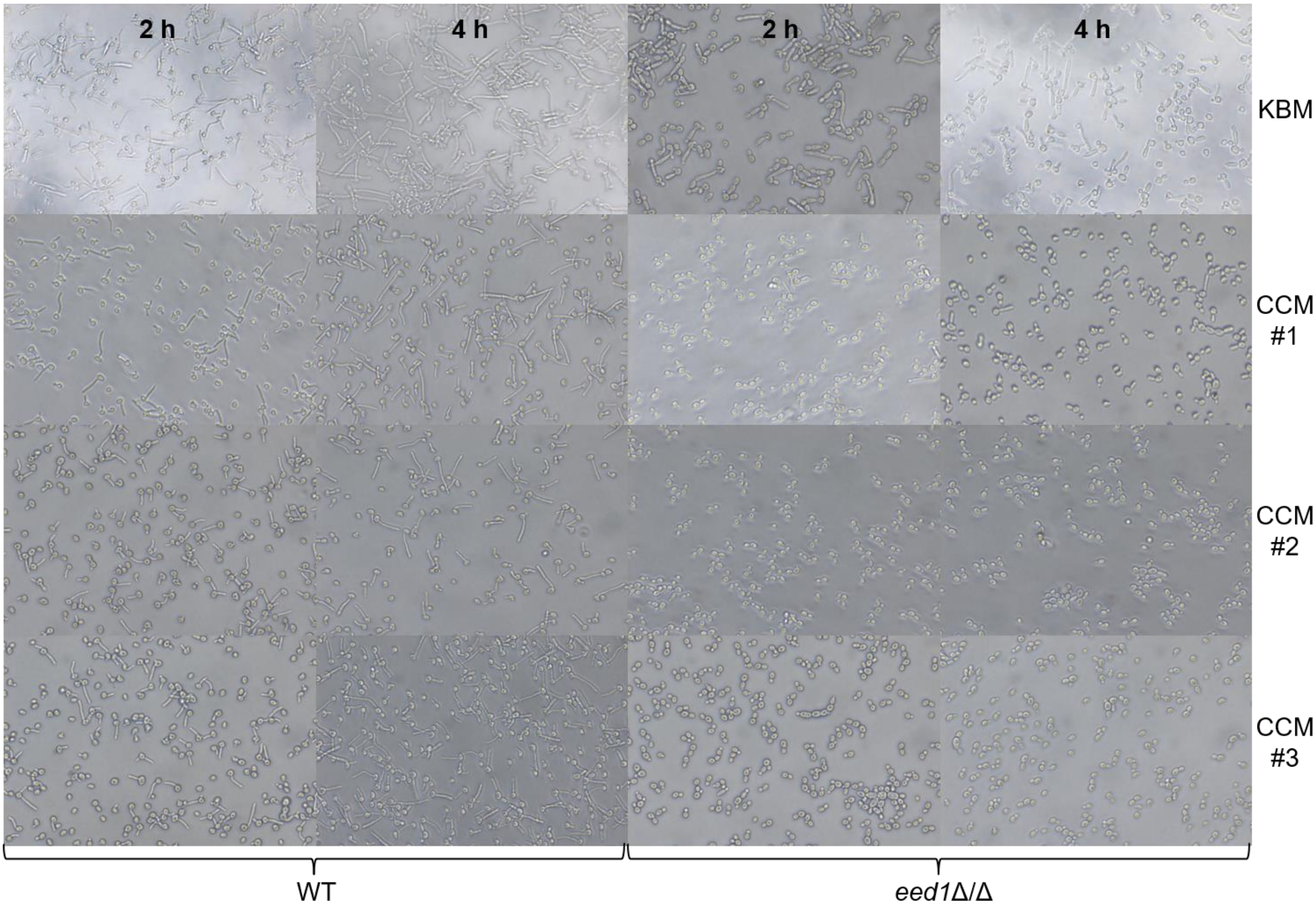

Figure S5: No filamentation of *eed1*Δ/Δ mutant cultivated in CCM

C. albicans SC5314 WT or eed1Δ/Δ were inoculated at 10^6 CFUs/ml into cell culture medium KBM or three independently generated CCM preparations and cultivated for 2 h and 4 h at standard cell culture conditions. After fixations, cells were investigated by light microscopy using a 10 × objective.
