# Supplementary material for: Augmented Enterocyte Damage During Candida albicans and Proteus mirabilis Coinfection
Source: Front Cell Infect Microbiol. 2022 May 16;12:866416. doi: 10.3389/fcimb.2022.866416 (PMC9149288; doi:10.3389/fcimb.2022.866416)
Supplement: Supplementary file 2 [file DataSheet_2.pdf]

Supplementary Table 1: List of fungal and bacterial strains used in this study

Microorganism	Reference/Source	Medium, temp	OD-CFU correlation# (CFUs/ml at OD_{600} 1); swarming on THB agar
Candida albicans BWP17-CIp30	[1]	YPD, 30 °C	N/A
Candida albicans BWP17-CIp30 *als3* Δ/Δ	[2]	YPD, 30 °C	
Candida albicans BWP17-CIp30 *ece1* Δ/Δ	[3]	YPD, 30 °C	
Candida albicans SC5314	NCBI:txid237561	YPD, 30 °C	
Candida albicans SC5314 *cap1* Δ/Δ	[4]	YPD, 30 °C	
Candida albicans SC5314 *eed1* Δ/Δ	[5]	YPD, 30 °C	
Candida albicans SC5314 *efg1*Δ/Δ	[6]	YPD, 30 °C	
Candida albicans SC5314 *efg1* Δ/Δ /*cph1* Δ/Δ	[7]	YPD, 30 °C	
Candida albicans SC5314 *hgc1* Δ/Δ	[8]	YPD, 30 °C	
Candida dubliniensis CD36	NCBI:txid573826	YPD, 30 °C	
Candida glabrata ATCC 2001	ATCC.org	YPD, 30 °C	
Candida parapsilosis DSM 4237	DSMZ.de	YPD, 30 °C	
Candida tropicalis DSM 4238	DSMZ.de	YPD, 30 °C	
Saccharomyces cerevisiae ATCC 9763	ATCC.org	YPD, 30 °C	
Proteus mirabilis BA6163	[9]	THB, 37 °C	1.60×10^{8} / yes
Proteus mirabilis DSM 4479	DSMZ.de	THB, 37 °C	1.09×10^{8} / yes
Proteus mirabilis DSM 788	DSMZ.de	THB, 37 °C	2.63×10^{8} / yes
Proteus mirabilis HI4320	[10]	THB, 37 °C	2.39×10^{8} / yes
Proteus mirabilis HI4320 *fliF*::kan	[11]	THB + 25 µg/ml kanamycin, 37 °C	2.52×10^{8} / no
Proteus mirabilis HI4320 *hpmA*::kan	[12]	THB + 25 µg/ml kanamycin, 37 °C	2.40×10^{8} / yes
Proteus mirabilis WPM111 (hmpA-negative)	[9]	THB + 25 µg/ml kanamycin, 37 °C	1.85×10^{8} / yes
Proteus mirabilis 101 (16-blod-411)	This study	THB, 37 °C	3.38×10^{8} / yes
Proteus mirabilis 102 (16-blod-6011)	This study	THB, 37 °C	3.72×10^{8} / yes
Proteus mirabilis 103 (16-blod-23290)	This study	THB, 37 °C	2.89×10^{8} / no
Proteus mirabilis 104 (16-blod-25869)	This study	THB, 37 °C	3.84×10^{8} / yes
Proteus mirabilis 105 (16-uri-29082)	This study	THB, 37 °C	3.30×10^{8} / no
Proteus mirabilis 106 (16-uri-29193)	This study	THB, 37 °C	4.19×10^{8}/ no
Proteus mirabilis 107 (16-uri-29207)	This study	THB, 37 °C	3.88×10^{8} / yes
Proteus mirabilis 108 (16-uri-29211)	This study	THB, 37 °C	3.47×10^{8} / no

The OD-CFU correlation was determined for all bacteria strains used in this study as follows: A subculture was grown to $OD_{600} \approx 1$. Cultures were then serially diluted in PBS and suitable dilutions were plated onto swarming-restricting CLED agar (#2835, Carl Roth). Colonies were counted after 18-24 h of incubation at 37°C. The mean of results from three independent experiments were used to calculate the OD-CFU correlation.

1. Wilson, D., et al., *Distinct roles of Candida albicans-specific genes in host-pathogen interactions.* Eukaryot Cell, 2014. **13**(8): p. 977-89.
2. Nobile, C.J., et al., *Critical role of Bcr1-dependent adhesins in C. albicans biofilm formation in vitro and in vivo.* PLoS Pathog, 2006. **2**(7): p. e63.
3. Moyes, D.L., et al., *Candidalysin is a fungal peptide toxin critical for mucosal infection.* Nature, 2016. **532**(7597): p. 64-8.
4. da Silva Dantas, A., et al., *Thioredoxin regulates multiple hydrogen peroxide-induced signaling pathways in Candida albicans.* Mol Cell Biol, 2010. **30**(19): p. 4550-63.
5. Martin, R., et al., *The Candida albicans-specific gene EED1 encodes a key regulator of hyphal extension.* PLoS One, 2011. **6**(4): p. e18394.
6. Lo, H.J., et al., *Nonfilamentous C. albicans mutants are avirulent.* Cell, 1997. **90**(5): p. 939-49.
7. Wartenberg, A., et al., *Microevolution of Candida albicans in macrophages restores filamentation in a nonfilamentous mutant.* PLoS Genet, 2014. **10**(12): p. e1004824.
8. Zheng, X., Y. Wang, and Y. Wang, *Hgc1, a novel hypha-specific G1 cyclin-related protein regulates Candida albicans hyphal morphogenesis.* EMBO J, 2004. **23**(8): p. 1845-56.
9. Mobley, H.L., et al., *Cytotoxicity of the HpmA hemolysin and urease of Proteus mirabilis and Proteus vulgaris against cultured human renal proximal tubular epithelial cells.* Infect Immun, 1991. **59**(6): p. 2036-42.
10. Pearson, M.M., et al., *Complete genome sequence of uropathogenic Proteus mirabilis, a master of both adherence and motility.* J Bacteriol, 2008. **190**(11): p. 4027-37.
11. Himpsl, S.D., et al., *Identification of virulence determinants in uropathogenic Proteus mirabilis using signature-tagged mutagenesis.* J Med Microbiol, 2008. **57**(Pt 9): p. 1068-1078.
12. Seo, S.U., et al., *Distinct Commensals Induce Interleukin-1beta via NLRP3 Inflammasome in Inflammatory Monocytes to Promote Intestinal Inflammation in Response to Injury.* Immunity, 2015. **42**(4): p. 744-55.

Supplementary Table 2: List of qPCR primers used in this study

**Gene**	**Forward (5'-3')**	**Reverse (5'-3')**
$hpmA_1$	ATCACTGGCTCTCAAGGTATTG	CTCAGAGCTCGCTGATTGTAG
$hpmA_2$	CACCACGCCAGAGAGTAAAG	CAATACCTTGAGAGCCAGTGATAG
hpmB	CGGCGTTGAATGGCTAAGT	CGATAGGGATAGGAGAGGGTATTG
rpoA	CACGTTATCTGCCACCTTACA	GATAGGGCGCTCATTCTTCTTC
secB	CCGCTTTCCTGTGTGAAGTA	TTCACGTGCATAAGGGAATAGT
cpxA	CATCTCAGAGCGACCGTATTT	CCACCTGGTCAACGTCTTATTA

Supplementary Table 3: Alterations of the pH in KBM, CCM and *Candida-Proteus* coinfections. At indicated time points of infections, the pH of all media was measured using a pH electrode. KBM and CCM previously chilled or frozen, were allowed to adjust to cell culture conditions (37 °C, 5 % CO_2) before measurement for at least 5 h. Values shown are means and SD of n independent samples.

Condition		pH (mean ± SD)	n (specification)
w/o enterocytes	KBM	7.44 ± 0.04	3 (bottles)
	CCM	7.49 ± 0.02	9 (independent batches)
w enterocytes	KBM 24 h	7.19 ± 0.02	3 (independent exp.)
	KBM 24+5 h	7.09 ± 0.03	
	C. albicans 24 h	7.23 ± 0.06	
	C. albicans 24+5 h	7.17 ± 0.02	
	KBM 24 h + *P. mirabilis* 5 h	6.97 ± 0.03	
	C. albicans 24 h + *P. mirabilis* 5 h	7.19 ± 0.02	
